# The Tudor Domain Protein Spindlin1 Is Involved in Intrinsic Antiviral Defense against Incoming Hepatitis B Virus and Herpes Simplex Virus Type 1

**DOI:** 10.1371/journal.ppat.1004343

**Published:** 2014-09-11

**Authors:** Aurélie Ducroux, Shirine Benhenda, Lise Rivière, O. John Semmes, Monsef Benkirane, Christine Neuveut

**Affiliations:** 1 Unité des Hépacivirus et Immunité Innée, Institut Pasteur, Paris, France; 2 UMR CNRS 3569, Paris, France; 3 The Leroy T. Canoles Jr Cancer Research Center and Department of Microbiology and Molecular Cell Biology, Eastern Virginia Medical School, Norfolk, Virginia, United States of America; 4 Institut de Génétique Humaine, CNRS UPR 1142, Laboratoire de Virologie Moléculaire, Montpellier, France; University of California, San Diego, United States of America

## Abstract

Hepatitis B virus infection (HBV) is a major risk factor for the development of hepatocellular carcinoma. HBV replicates from a covalently closed circular DNA (cccDNA) that remains as an episome within the nucleus of infected cells and serves as a template for the transcription of HBV RNAs. The regulatory protein HBx has been shown to be essential for cccDNA transcription in the context of infection. Here we identified Spindlin1, a cellular Tudor-domain protein, as an HBx interacting partner. We further demonstrated that Spindlin1 is recruited to the cccDNA and inhibits its transcription in the context of infection. Spindlin1 knockdown induced an increase in HBV transcription and in histone H4K4 trimethylation at the cccDNA, suggesting that Spindlin1 impacts on epigenetic regulation. Spindlin1-induced transcriptional inhibition was greater for the HBV virus deficient for the expression of HBx than for the HBV WT virus, suggesting that HBx counteracts Spindlin1 repression. Importantly, we showed that the repressive role of Spindlin1 is not limited to HBV transcription but also extends to other DNA virus that replicate within the nucleus such as Herpes Simplex Virus type 1 (HSV-1). Taken together our results identify Spindlin1 as a critical component of the intrinsic antiviral defense and shed new light on the function of HBx in HBV infection.

## Author Summary

Hepatitis B virus (HBV) represents a major risk factor for the development of hepatocarcinoma. Within the nucleus, HBV transcription is activated by both cellular and viral factors but is also repressed by cellular proteins that could be part of cellular antiviral defense mechanisms. Recently it has been shown that the regulatory protein HBx is essential to initiate and maintain HBV transcription in the setting of infection. Here we identify Spindlin1, a cellular protein involved in transcriptional regulation, as an HBx interacting partner. We show that Spindlin1 is recruited to the HBV DNA and inhibits its transcription in the context of infection. A virus deficient for the expression of HBx is more severely repressed by Spindlin1 than the wild type virus, suggesting that HBx counteracts Spindlin1 repression. We found that Spindlin1 also represses the transcription of Herpes Simplex Virus type 1 in the setting of infection. Our study not only provides new insights into the mechanisms regulating HBV transcription and the role of HBx in this process, but also reveals a new function of Spindlin1 as a component of the intrinsic antiviral defense.

## Introduction

Despite the existence of an effective preventive vaccine, hepatitis B virus infection remains a major health problem. Chronic HBV infection affects 350 million people worldwide who are at high risk of developing liver diseases including cirrhosis and hepatocellular carcinoma (HCC) [1]. HBV is a prototypical member of the hepadnavirus family of DNA viruses that preferentially target hepatocytes and share the particularity to replicate their genome via an RNA intermediate. The virion consists of a 3.2 kb partially double stranded relaxed circular DNA (RC-DNA). Upon infection, RC-DNA is delivered into the nucleus and converted into a covalently closed circular DNA (cccDNA) that serves as the template for transcription of all viral RNAs including the pregenomic RNA (pgRNA). PgRNA is then encapsidated in the cytoplasm and retrotranscribed into RC-DNA. Capsids containing RC-DNA are either enveloped at the endoplasmic reticulum and released from the cell or are recycled to the nucleus and contribute to the amplification of cccDNA.

cccDNA is organized into a chromatin like structure and viral genes transcription is directed by four promoters and two enhancers. cccDNA transcription is likely regulated as cellular DNA by the activity of transcription factors, histone modifiers and chromatin remodelers [2], [3], [4], [5], [6], [7], [8]. The importance of chromatin in the regulation of HBV expression is supported by studies showing that HBV transcription correlates with the hyperacetylation of histone H3 and H4 and the recruitment of the coactivators CBP/p300 [6]. On the contrary, HBV silencing correlates with the deacetylation of H3 and H4 and the recruitment of HDAC1 and Sirt1 [6]. Relevant with these finding we showed that knockdown of CBP and P300 reduced HBV transcription [4]. Finally, interferon-α represses HBV transcription via epigenetic mechanisms involving the recruitment of the chromatin remodeling complex Polycomb Repressive Complex 2 (PRC2) to cccDNA [9].

Beside cellular factors, the regulatory protein HBx that is essential for virus replication plays a crucial role in HBV transcription [10], [11], [12]. A study from Lucifora and colleagues has shown that HBx is required for the initiation and the maintenance of HBV RNA transcription during infection [13]. They showed that in this setting, the expression of HBx correlates with the hyperacetylation of histone H3 associated to the HBV wt cccDNA arguing that HBx might favor HBV transcription through the modulation of epigenetic marks. This finding is in agreement with studies showing that the recruitment of HBx to the cccDNA correlates with the recruitment of its interacting partners CBP, P300 and PCAF, with histone H3 acetylation and with HBV transcription [10], [14]. Moreover, HBx activates HBV transcription through the inhibition of cellular factors involved in chromatin regulation such as the PP1/HDAC1 complex and PRMT1 [2], [4]. While HBx activates cellular genes and HBV transcription via specific target sequences such as the cyclic AMP-response element (CRE), Breugel and collaborators have recently suggested that HBx also increases the transcriptional activity of enhancers and promoters DNA regardless of their sequence when they are located on extrachromosomal DNA [15]. They thus hypothesize that HBx might either recruit to episomal DNA factors necessary for transcription such as coactivator or destabilized a restriction factor that recognizes and silences foreign DNA. Further studies are needed to understand the mechanism of HBV silencing and uncover these restrictions factors. Moreover, it will be necessary to determine whether plasmid DNA and cccDNA are silenced through the same mechanisms.

Spindlin1 was initially identified in mice as a highly expressed maternal protein that might play a role during meiosis [16]. Its human homologue that is overexpressed in different tumors tissue including ovarian cancer has been shown to induce cell-cycle-delay in metaphase and chromosome instability [17], [18], [19]. While its functions in the cell remain largely unknown, recent finding suggest that Spindlin1 is involved in transcriptional regulation. Spindlin1 is structured in three Tudor-like domains in tandem [20], [21]. Tudor domains are conserved motifs that are involved in the recognition and binding to methylated lysine or arginine of target substrates, facilitating the assembly of protein complexes [22], [23]. Spindlin1 has been shown to interact with trimethylated lysine 4 of histone H3 (H3K4me3) and to stimulate ribosomal RNA transcription [24]. Spindlin1-H3K4me3 interaction is mediated by the second Tudor-like domain of Spindlin1 [20], [24]. Finally, it has been recently demonstrated that Spindlin1 simultaneously recognizes H3 asymetric dimethylarginine 8 and H3K4me3 on the promoter of Wnt target genes via its Tudor-like domains 1 and 2 respectively and activates transcription. All together, these data suggest that Spindlin1 is involved in transcriptional regulation through histone post-translational modifications (PTM) sensing [25]. However, the second Tudor-like domain of Spindlin1 is also involved in the recognition of the cellular serpine mRNA binding protein 1 (SERBP1) and in the formation of a complex involved in the regulation of mRNA stability and/or translation [26]. Thus Spindlin1 via the interaction with cellular proteins might be involved in different cellular activities.

In this study, we show that Spindlin1 represses HBV transcription in the context of infection. We demonstrate that Spindlin1 is recruited to the HBV cccDNA and that repression is dependent on its tudor-like domain II. Finally, Spindlin1 repression extends to HSV-1 transcription during infection of HepaRG hepatoma cells. Together our findings suggest that Spindlin1 participates in the intrinsic antiviral defense against at least two DNA viruses, HBV and HSV-1.

## Materials and Methods

### DNA plasmids and siRNA

Wild type HBx (adw subtype) and deletion mutants carrying an N-terminal HA tag have been described previously [14]. HBx clustered alanine substitution mutants: HBx Cm5, HBx Cm7, HBx Cm8, HBx Cm9 and HBx Cm13 as well as Flag-HBx wt were kindly provided by S. Murakami and has been described previously [12]. The expression vector for His-Myc-Spindlin1 was provided by W. Yue [19]. Full-length Flag-Spindlin1 and the mutants Flag-Spindlin1-Y170A and Flag-Spindlin1-F141A were kindly provided by B. Zhu [24].

His-Myc-Spin1mut plasmid encoding Spindlin1 deleted of the second Tudor like domain, was generated by several PCR amplifications using the His-Myc-Spindlin1 expression vector [19]. PCR1 was performed using the following primers: sense 5′GCGGCCGCCACTGTGCTGGATATCTGCAGA3′ containing the restriction site EcoRV and anti-sense 5′TGAATCATTGGAATCAGGTTTGCCAATCATTGTGTCTGC3′ and PCR2 was performed using the following primers: sense 5′ TTCGGGCCCAAGCTTGGTACCGAGCTCGGA3′and anti-sense 5′GCAGACACAATGATTGGCAAACCTGATTCCAATGATTCA3′ containing the restriction site HindIII. The products of PCR1 and PCR2 were then mixed and amplified by PCR using the following primers: sense 5′GCGGCCGCCACTGTGCTGGATATCTGCAGA3′ and anti-sense 5′GCAGACACAATGATTGGCAAACCTGATTCCAATGATTCA3′ containing the restriction site EcoRV and HindIII respectively. The EcoRV-HindIII fragment containing the cDNA Spin1mut was cloned in His6-Myc-Spindlin1 digested by EcoRV-HindIII. All the constructions have been verified by sequencing.

Control siRNAs and siGENOME SMART pool siRNAs directed against Spindlin1 were purchased from ThermoScientific:

SPIN1 D-019983-17: GCAUGCCAGUGAAGCGUUA

SPIN1 D-019983-18: CCACAUGUAAUGACGCUAU

SPIN1 D-019983-04: GUUGCGACAUCUCGAAUCA

SPIN1 D-019983-02: GGACUAGAACUUAAUAAAG

### Cell culture, DNA and siRNA transfections

HEK293 and HEK293T cells were maintained in Dulbecco's modified Eagle's medium (DMEM) (Gibco) with 10% fetal bovine serum (FBS). Huh7.25.CD81 has been described previously and was maintained in DMEM (Gibco) with 10% FBS [27]. HepG2, HepG2.2.15, HepG2 H1.3Δx and HepAD38 cells were maintained in DMEM-F12 complemented with 10% FBS, 3.5×10^−7^ M hydrocortisone hemisuccinate, and 5 µg/ml insulin. HepAD38 cell line is derived from HepG2 cells and contains the HBV genome under tetracycline control [28]. HepG2 H1.3Δx (HBV X-) cells are derived from the HepG2 cell line and contain the stable integration of respectively a 1.3-fold HBV genome (genotype D, subtype ayw) or a 1.3-fold HBV genome carrying premature stop codon mutations in both the 5′ and 3′ HBx open reading frames [13]. HepaRG cells were maintained in William's E medium (Gibco) supplemented with 5% fetal calf serum, 7×10^−5^ M hydrocortisone hemisuccinate, 5 µg/ml insulin and 2% DMSO (Sigma-Cell Culture reagent). HepaRG differentiation has been described previously [29]. Briefly cells were maintained for two weeks in standard medium then for at least 2 weeks in standard medium supplemented with 1,8% DMSO and EGF (5 ng/ml) (PeproTech-Tebu France). Cells were transfected with different vectors as indicated in the Figure legends. For immunoprecipitation, HEK293 cells were transfected 24 h after seeding using SiImporter reagent (Millipore) according to the manufacturer's protocol. Total amounts of transfected DNA were kept constant by adding empty vector DNA. HepAD38 cells were grown without tetracycline and electroporated with Spindlin1 wild type or the mutants (Y170A, F141A) with nucleofection technology according to the manufacturer's protocol (Amaxa). HepAD38 shCtrl or shSpindlin1 cells were grown without tetracycline and transfected with 20 nM of siCtrl1 or siSpindlin1 respectively using Interferin reagent according to the manufacturer's protocol (PolyPlus transfection).

### Lentiviral vectors, shRNA and transduction

pTRIP-Flag-HA-HBx plasmid has been previously described [2]. pTRIP-His-Myc-Spindlin1 plasmid was generated by PCR amplification from the His-Myc-Spindlin1 expression vector [19] using the following primers: sense 5′CCGGGGATCCATGATGAAGAAGAGGACAT3′ and antisense 5′CCGGCTCGAGTCAATGATGATGATGATGATGGC3′ containing the restriction sites BamHI and XhoI. pTRIP-His-Myc-Spindlin1 plasmid was generated by cloning the BamHI-XhoI fragment containing His-Myc-Spindlin1 cDNA in the BamHI-XhoI sites of the lentiviral vector pTRIPΔU3. pTRIP Flag-HA (Mock) and pTRIP His-Myc (Mock) contain, respectively the Flag-HA and the His-Myc Tag cloned into the lentiviral vector pTRIPΔU3. Virions were produced by calcium phosphate transfection of HEK293T cells as previously described [14]. Supernatants were collected 3 days after transfection and virus were purified by ultracentrifugation through a 20% (wt/vol) sucrose cushion. Virus production was normalized by measuring supernatant reverse transcriptase (RT) activity. Transduction of HepG2, HepAD38 or HepaRG cells was performed using normalized viruses. Virus were first absorbed in a small volume for 2 h at 37°C. Fresh medium was then added, and cells were incubated for 2 days.

pALPS-shSpindlin1.1, pALPS-shSpindlin1.2 and pALPS-shSpindlin1.3 plasmids were generated by PCR amplification from the following template:

**shSpindlin1.1:**

5′TGCTGTTGACAGTGAGCGATGGAACTATGAAATGTATTATTAGTGAAGCCACAGATGTAATAATACATTTCATAGTTCCACTGCCTACTGCCTCGGA3′

**shSpindlin1.2:**

5′TGCTGTTGACAGTGAGCGCGCCCTCCGTCTATTTCATCAATAGTGAAGCCACAGATGTATTGATGAAATAGACGGAGGGCTTGCCTACTGCCTCGGA3′

**shSpindlin1.3:**

5′TGCTGTTGACAGTGAGCGACCAAATTTGTGGAACTATGAATAGTGAAGCCACAGATGTATTCATAGTTCCACAAATTTGGCTGCCTACTGCCTCGGA3′

with the following primers:

**sense** 5′GATGGCTGCTCGAGAAGGTATATTGCTGTTGACAGTGAGCG3′

**antisense** 5′GTCTAGAGGAATTCCGAGGCAGTAGGCA3′ containing the restriction sites XhoI and EcoRI. The XhoI-EcoRI fragment containing the shRNAs against Spindlin1 were cloned in the XhoI-EcoRI sites of the lentiviral vector pALPs-mir30. Virions were produced as a pool as described previously. HepAD38 or HepaRG cells stably expressing shSpindlin1 or the control shCtrl were established by transducing cells twice with virus normalized for RT activity. After 48 h of incubation with fresh medium, clones have been selected with puromycine (4 µg/ml). The pool of selected clones was analyzed for Spindlin1 expression by Western blot and quantitative RT-PCR (RT-qPCR).

### Antibodies and reagent

ActinomycinD was purchased from Sigma-Aldrich and used at a concentration of 5 µg/ml. The protease inhibitors were obtained from Roche (Complete EDTA free) and from Sigma-Aldrich (Pefabloc). TURBO DNAse was purchased from Ambion (Life technologies). Antibodies are described in Table 1.

**Table 1:** Antibodies.

Antibody	Reference		Application
Flag M2	Sigma Aldrich	F3165	IP/Immunoblot
GAPDH	Abcam	ab 8245	Immunoblot
IgG	Millipore	PP64B	ChIP
H3K4me3	Millipore	07-473	ChIP
HA	Covance	MMS 101R	Immunoblot
His	Novagen	71841	Immunoblot
Myc	Calbiochem	OP10	Immunoblot
Spindlin1	Proteintech	12105-1-AP	ChIP/IP/Immunoblot
Spindlin1	Abcam	ab118784	Immunoblot
Tubulin	Sigma	T5168	Immunoblot
β-catenin	BD Biosciences	610153	Immunoblot

### Immunoprecipitation and western blot

For immunoprecipitation and Western blot experiments, cells were lysed at 4°C in lysis buffer (400 mM KCl, 20 mM Tris pH 7,5, 5 mM MgCl_2_, 0,1% Triton X-100, 0,5 mM EDTA, 10% glycerol, 10 mM de β-mercaptoethanol, 0.5 mM PMSF) containing EDTA-free protease inhibitors cocktail (Roche). After lysis, the extracts were cleared by centrifugation (4°C, 13000 rpm, 20 min). Supernatant was then incubated with appropriate antibodies and beads for 2 h. Proteins complexes bound to the beads were washed 3 times in lysis buffer and then eluted from the beads by boiling in Laemmli buffer for 10 min. Samples were resolved by SDS-PAGE and electro-transferred to nitrocellulose membranes. Blots were incubated with the indicated primary antibodies and then with alkaline phosphatase conjugated secondary antibodies. Proteins were visualized by chemiluminescence (TROPIX, Applied Biosystem). When using Odyssey procedure, after incubation with the primary antibody, blots were probed with Dye-conjugated secondary antibodies. Fluorescent immunoblot images were acquired and quantified by using an Odyssey scanner and Odyssey 3.1 software (Li-CorBiosciences).

### Virus production and infection

For HBV particles production, HepAD38 (HBV WT) or HepG2 H1.3Δx (HBV X-) cells were cultivated in Williams E medium containing 5% fetal calf serum 7×10^−5^ M hydrocortisone hemisuccinate, 5 µg/ml insulin and 1% DMSO. HBV particles were concentrated from the clarified supernatant of the cells by overnight precipitation with 5% PEG 8000 and centrifugation at 4°C for 60 min at 5000 rpm. The pellet was resuspended with complete William's medium supplemented with 2% DMSO. Enveloped DNA-containing viral particles were tittered in the HBV inocula by immunoprecipitation with an anti PreS1 antibody followed by qPCR quantification of viral RC DNA with the primers RC 5′ and RC 3′ (Table 2) [4]. Only enveloped DNA-containing viral particles were taken into account when the multiplicity of infection (MOI) was calculated. Differentiated HepaRG cells were infected as described with normalized amounts of virus at the indicated MOI [13].

**Table 2:** Primers.

Primers	Sequences	Experiment
HBV RT sense	5′-GCTTTCACTTTCTCGCCAAC-3′	RT-qPCR
HBV RT anti-sense	5′-GAGTTCCGCAGTATGGATCG-3′	RT-qPCR
Roth2 sense	5′-CTGCGGACTATCTCTCCCCTC-3′	RT-qPCR
Roth2 anti-sense	5′-AAAAGGCTTTGCAGCTCCAC-3′	RT-qPCR
Spindlin1 sense	5′-GCCATGCTGGAGTATCTGC-3′	RT-qPCR
Spindlin1 anti-sense	5′-AGAACGGTTCCTTTCCACTG-3′	RT-qPCR
pre-ARNr sense	5′-TGTCAGGCGTTCTCGTCTC-3′	RT-qPCR
pre-ARNr anti-sense	5′-AGCACGACGTCACCACATC-3′	RT-qPCR
ICP27 sense	5′-GCATCCTTCGTGTTTGTCATT-3′	RT-qPCR
ICP27 anti-sense	5′-GCATCTTCTCTCCGACCCCG-3′	RT-qPCR
HCV sense	5′-TGCGGAACCGGTGAGTACA-3′	RT-qPCR
HCV anti-sense	5′-CGGGTTGATCCAAGAAAGGA-3′	RT-qPCR
rDNA sense	AGTCGGGTTGCTTGGGAATGC	ChIP-qPCR
rDNA anti-sense	CCCTTACGGTACTTGTTGACT	ChIP-qPCR
HBV cccDNA sense	5′-GTGCACTTCGCTTCACCTCT-3′	ChIP-qPCR
HBV cccDNA anti-sense	5′-AGCTTGGAGGCTTGAACAGT3′	ChIP-qPCR
CCNA2 sense	5′-CCTGCTCAGTTTCCTTTGGT-3′	ChIP-qPCR
CCNA2 anti-sense	5′-AGACGCCCAGAGATGCAG-3′	ChIP-qPCR
RC sense	5′-CACTCTATGGAAGGCGGGTA-3′	IP preS1-qPCR
RC anti-sense	5′-TGCTCCAGCTCCTACCTTGT-3′	IP preS1-qPCR
CCNA2 ARN sense	5′- CTCCAAGAGGACCAGGAGAA-3′	RT-qPCR
CCNA2 ARN anti-sense	5′- TGAACGCAGGCTGTTTACTG-3′	RT-qPCR

HSV-1 strain KOS was propagated in U373MG cells and was kindly provided by M. Lafon [30]. Briefly, 1.10^6^ undifferentiated HepaRG cells were incubated at 37°C for 1 h with virus at the indicated MOI. After 1 hour of incubation, cells were washed with DMEM, centrifuged for 5 min at 1500 rpm and then cultured in 6-well plates for the indicated time.

HCV JFH1 was propagated in Huh7.25/CD81 cells was kindly provided by E. Meurs. The preparation of JFH1 stocks have been described previously [27].

### cccDNA extraction

cccDNA was purified from differentiated HepaRG cells 8 days after infection with HBV wild-type or HBV X- viruses. Cells were harvested and lysed in buffer 1 (10 mM Hepes pH 8, 1.5 mM MgCl 2, 10 mM KCl, 1 mM DTT, 0.5 mM pefablock, EDTA-free complete protease inhibitors [Roche Applied Science]). After 15 min at 4°C, NP40 1% was added. Supernatant (cytosol) and pellet (nuclei) were separated by centrifugation (2000 rpm, 10 min, 4°C). Nuclear content was extracted in a volume/volume mixture of buffer A (50 mM KCl, 10 mM Tris pH 8.3, 2.5 mM MgCl2) and buffer B (0.5% NP40, 0.5% Tween, 2.5 mM MgCl 2). Proteinase K (Fermentas) digestion was then carried out for 1 h at 45°C. After DNA purification, cccDNA was quantified by qPCR using specific primers (Table 2). Level of Cyclin A2 gene (CCNA2) was used for normalization.

### Genomic HSV1 DNA extraction

Genomic DNA of HSV1 was extracted from HepaRG cells 2 h post infection. Cells were incubated 15 min at 37°C in lysis buffer (50 mM Tris-HCl pH 8, 10 mM EDTA, 150 mM NaCl, 1% SDS). KCl 0.625 M was added and the supernatant was recovered after centrifugation at 13,000 rpm for 15 min. DNA was purified by phenol-chloroform extraction and genomic HSV1 DNA was quantified by qPCR using specific primers (Table 2). The level of CyclineA2 gene was used for normalization.

### Chromatin immunoprecipitation (ChIP)

ChIP experiments were carried out on infected HepaRG cells 8 days post infection as described previously with minor modifications [2]. Briefly, cells were fixed with 1% formaldehyde for 10 min at 37°C and quenched with 0.125 M Glycine for 2 min at room temperature. After washing the cells in PBS 1×, nuclear extracts were prepared as follows: cells were lysed for 10 min at 4°C in buffer A (0.25% triton X-100, 10 mM Tris pH8, 0.5 mM pefablock, EDTA-free protease inhibitors (Roche)) and centrifuged at 1500 rpm for 5 min at 4°C. Nuclei were washed for 10 min at 4°C in buffer B (0.2 M NaCl, 10 mM Tris pH8, 0.5 mM pefablock, EDTA-free protease inhibitors), centrifuged and lysed in nuclei lysis buffer (1% SDS, 10 mM EDTA, 50 mM Trip pH8, 0.5 mM pefablock, EDTA-free protease inhibitors). Lysates were then sonicated in a Bioruptor sonication device (Diagenode) for 10 min (pulses of 30 s). After centrifugation, supernatants were diluted 1∶10 with dilution buffer (0.01% SDS, 1.1% Triton X-100, 1.2 mM EDTA, 16.7 mM Tris pH8, 167 mM NaCl 0.5 mM pefablock, EDTA-free protease inhibitors). Chromatin was then subjected to immunoprecipitation overnight at 4°C using 2 µg of indicated primary antibody. Immunoprecipitations with relevant nonspecific immunoglobulins (Millipore, PP64B) were included in each experiment as a negative control. Immune complexes were incubated with 40 µl of a mix of protein A/protein G agarose beads (Santa Cruz Biotechnology) for 1 h at 4°C. The immunoprecipitates were washed five times in RIPA buffer containing 0.5 mM pefablock and EDTA-free protease inhibitors (Roche), and twice in TE buffer and then eluted in elution buffer (1% SDS, 0.1% NaHCO3). After purification of the immunoprecipitated DNA, qPCR was performed using primers that amplify with higher specificity the cccDNA (HBV cccDNA sense and HBV cccDNA anti-sense) or using specific primers for the rDNA (Table 2). Samples were normalized to input DNA using the ΔCt method were ΔCT = CT (input)-CT (immunoprecipitation) and calculated as percentage of the input. Results are expressed as the average of at least three independent experiments. Standard deviations are indicated.

### Quantitative RT-PCR

Total RNA was prepared from transfected HepAD38 cells or infected HepaRG cells using TRIzol reagent (Invitrogen) and TURBO DNA-free reagent (Ambion). RNA (500 ng) was retrotranscribed using random primers and RevertAid H Minus M-MuLV reverse transcriptase (Fermentas). cDNA was analyzed by quantitative PCR (qPCR) using SybrGreen PCR Master mix (Applied Biosystems) on ABI PRISM 7900HT Sequence Detection System (Applied Biosystems) using standard PCR protocol (denaturation at 95°C and annealing/extension at 63°C), and a final dissociation step to ensure amplicon-specific detection. The primers used in RT-qPCR are listed in table 2. The primers HBV RT sense and HBV RT anti-sense amplify all HBV transcripts except the 0.8 kb transcript encoding HBx. Roth2 was used as a reference gene because of its low variation coefficient in human liver tumors and cell lines [31]. All assays were performed in triplicate using 0.5 µl of cDNA per reaction and mean values were calculated according to the ΔCT quantification method. Results are expressed as the average of at least three independent experiments. Standard deviations are indicated. Statistical differences were analyzed using the non-parametric Wilcoxon signed-rank test for paired data when more than five experiments were included in the analysis.

#### Primers for qChIP, Quantitative PCR and Quantitative RT-PCR

All synthetic oligonucleotides were ordered from Eurofins MWG Operon (Table 2).

### Replication intermediates extraction and Southern blot

HBV replication intermediates were extracted from HepAD38 cells control or expressing His-Myc-Spindlin1. 48 h after transduction, cells were harvested and lysed in lysis buffer (100 mM Tris-HCl pH 8, 0.2% NP40). After 1 min of centrifugation at 13,000 rpm, the supernatant was recovered, and 3.3 mM MgOAc was added. The samples were then digested with 100 µg DNAse I and 50 µg RNAse A (Roche) for 1 h at 37°C. After 1 min of centrifugation at 13,000 rpm, the supernatant was collected and supplemented with 6.25 mM EDTA, 0.5% SDS, 62.5 mM NaCl and 150 µg of proteinase K. After 1 h at 55°C, the DNA was purified. After 1 h at 55°C, HBV replication intermediates were recovered by phenol-chloroform extraction and isopropanol precipitation. DNA was analyzed by electrophoresis through 1% agarose gel and transferred to nylon membranes (Amersham Hybond-XL). Blots were hybridized with ^32^P-labeled full-length HBV genome. Relative intensities of DNA RC and SS DNA were quantified using the Storm840 PhosphorImager (Molecular Dynamics).

For analysis of cccDNA by Southern blot, total DNA was extracted as described previously [32]. Briefly cells were lysed in lysis buffer (50 mM Tris pH8.0; 10 mM EDTA; 150 mM NaCl; 1% SDS) at 37°C for 1 hour. Cellular DNA and cccDNA were separated from replicative viral DNA (relaxed circular DNA and single stranded DNA) by precipitation with 0.6 M KCl. After precipitation cellular DNA and cccDNA were recovered from the supernatant by phenol/chloroform extraction and ethanol precipitation. 50 µg of DNA were resolved by electrophoresis through 1% agarose gel and transferred to nylon membranes (Amersham hybond-XL). Blots were hybridized with ^32^P-labelled full-length HBV genome.

### Northern blot analysis

Total RNA was extracted using TRIzol reagent as recommended by the manufacturer (Invitrogen). RNA samples (20 µg) were resolved on 1% formaldehyde-agarose gel and transferred to a Hybond N+ nylon membrane (Amersham). Blots were hybridized with full-length 3.2 kb HBV DNA or 18S rDNA probes labeled by random priming.

### Nuclear run-on

Nuclei were isolated from Hepa38 cells and the reaction was performed as previously described [33]. Briefly, HepAD38 cells were incubated for 5 min at 4°C with a cold hypotonic buffer (10 mM Tris-HCl pH 7.5, 2 mM MgCl2, 3 mM CaCl2). After centrifugation at 1000 rpm for 10 min, the pellet was resuspended in lysis buffer (10 mM Tris-HCl pH 7.5, 2 mM MgCl2, 3 mM CaCl2, 0,5% NP40, 10% glycerol, RNAse inhibitor [Promega]). Nuclei were then washed 1 time with lysis buffer and once with freezing buffer (50 mM Tris -HCl, 40% glycerol, 5 mM MgCl2, 0.1 mM EDTA, RNAse inhibitor [Promega]). Nuclei were added to reaction buffer (10 mM Tris -HCl pH 8, 5 mM MgCl2, 1 mM DTT, 300 mM KCl, RNAse inhibitor [Promega]), containing ATP (TebuBio), CTP (TebuBio), GTP (TebuBio), and Br-UTP (Invitrogen) (500 µM each) in the presence of 0,5% sarkosyl. After incubation at 31°C for 25 min, total RNA was extracted using the Trizol reagent (Invitrogen) according to the manufacturer's recommendation and treated with DNase (TURBO DNase-free, Ambion). Denatured RNAs (90°C, 5 min) were then incubated with agarose beads conjugated with anti-BrU antibodies. After1 h at 4°C under agitation, beads were washed 1 time with binding buffer (0,25× SSPE, 1 mM EDTA, 0,05% Tween 20, 37,5 mM NaCl), 1 time with a low concentration salt buffer (0.2× SSPE, 1 mM EDTA, 0.05% Tween 20), 1 time with a high concentration salt buffer (0.25× SSPE, 1 mM EDTA, 0.05% Tween 20, 100 mM NaCl) and 2 times with TET buffer (TE, 0.5% Tween 20) buffer. All washes were carried out at 4°C. Four successive elutions were performed with elution buffer heated to 42°C (20 mM DTT, 150 mM NaCl, 50 mM Tris pH 7.5, 1 mM EDTA, 0.1% SDS). The eluates were then combined and labeled purified RNAs were analyzed by RT-qPCR using the primers HBV RT and Roth2 (table 2).

## Results

### Spindlin1 interacts with HBx

**Figure 1:**
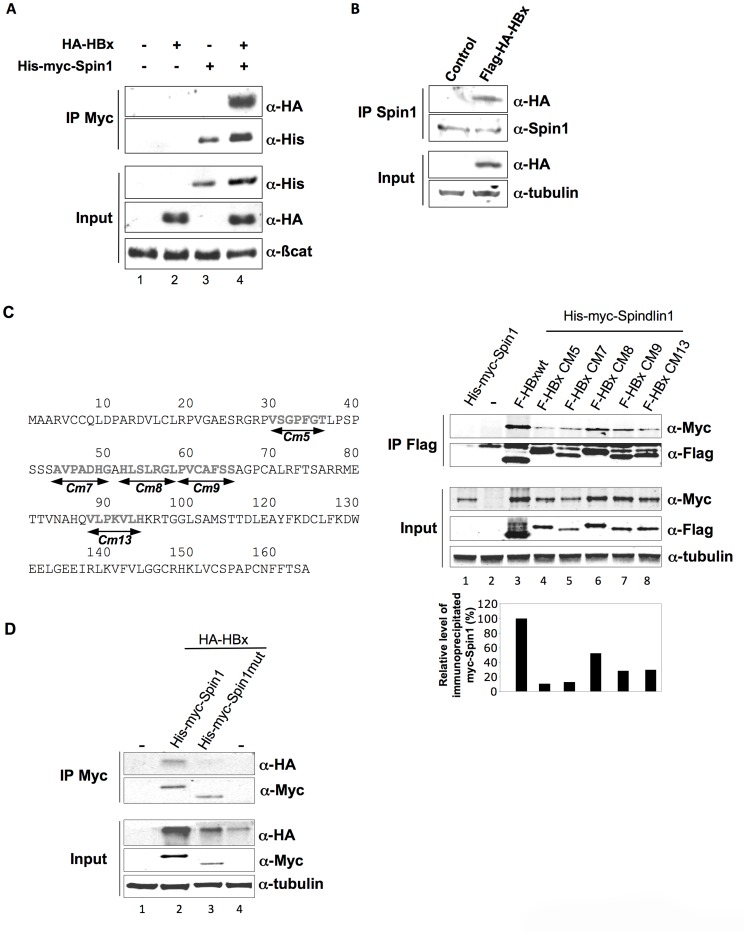
Spindlin1 interacts with HBx. (**A**) Coimmunoprecipitation of His-myc-Spindlin1 (His-myc-Spin1) with HA-HBx using anti-Myc antibodies in HEK293 cells. Proteins in the immune complexes were revealed by Western blotting with anti-His and anti-HA antibodies. The expression of β-catenin was used as loading control. (**B**) Whole-cell extracts, prepared from HepG2 cells transduced with a lentiviral vector encoding Flag-HA-HBx, were immunoprecipitated with anti-Spindlin1 antibodies (IP Spin1). Proteins were detected by Western blotting using anti-HA or anti-Spin1 antibodies. The expression of tubulin was used as loading control. (**C**) Schematic representation of the HBx clustered alanine substitution mutants (left panel). HEK293 cells were co-transfected with Flag-tagged HBx wt construct or the HBx alanine substitution mutants (F-HBx Cm5, F-HBx Cm7, F-HBx Cm8, F-HBx Cm9, F-HBx Cm13) and the His-myc-Spin1 plasmid. Cell extracts were immunoprecipitated with anti-Flag antibodies and analyzed by anti-Myc and anti-Flag immunoblot using the Odyssey system (right panel). The expression of tubulin was used as loading control. Signal strengths of the co-immunoprecipitated His-myc-Spindlin1 proteins were normalized to the ratio of HBx in the IP/HBx in the input. Immunoprecipitated His-myc-Spindlin1 level in cells expressing His-myc-Spindlin1 and Flag-HBx wt was set to 100% (lower right graph) (**D**) Extracts from HEK293 cells transfected with HA-HBx in combination with His-Myc-Spin1 or His-myc-Spindlin1 mutant containing a deletion of the Tudor-like domain II (His-myc-Spin1 mut) were immunoprecipitated with anti-Myc antibodies. Proteins were detected by Western blotting using anti-HA or anti-Myc antibodies. The expression of tubulin was used as loading control.

It has been shown that HBx is required for the establishment of a transcriptionally active cccDNA during infection [13]. Recently, we identified using tandem affinity purification technique several cellular interacting partners for HBx. In a first study, we have focused on those known to be involved in chromatin regulation and demonstrated that HBx can interfere with their function to the benefit of HBV transcription [2]. Among the cellular interacting partners for HBx we also identified Spindlin1, a protein that is involved in transcriptional regulation, although its exact function is not clear. Spindlin1 transcriptional activity is linked in part to its ability to read histone modifications [24], [25]. Since HBV transcriptional activation by HBx relies on the modulation of histones post-translational modifications, we further studied whether Spindlin1 is involved in HBV transcriptional regulation [10], [13]. The ability of HBx to interact with Spindlin1 was first confirmed by immunoprecipitation assay using cellular extract from HEK293 cells transfected with His-Myc-Spindlin1 (His-myc-Spin1) and HA-HBx vectors (Figure 1A). We next used cellular extracts of HepG2 cells transduced with a lentiviral vector coding for Flag-HA-HBx or the control vector. We showed, using anti-Spindlin1 antibodies that HBx coimmunoprecipitates with endogenous Spindlin1 (Figure 1B). Finally we searched for the domain of HBx involved in the interaction with Spindlin1. HEK293 cells were first transfected with in frame deletion mutants of HBx fused to HA tag at the N-terminus together with His-Myc-Spindlin1 vectors (Figure S1, left panel). Co-immunoprecipitation assays using anti-HA antibodies revealed that binding of HBx with Spindlin1 was lost the for ×5 and ×6 mutants. This clearly indicates that amino acids 1 to 58 of HBx are required for the interaction with Spindlin1. Furthermore, a weaker interaction was also observed between the ×9 mutant and Spindlin1, suggesting that the domain spanning amino acids 84 to 120 is also involved in Spindlin1 interaction (Figure S1, right panel). To further confirm the role of these two regions, we then performed immunoprecipitation experiments using Flag-tagged HBx mutants containing clustered alanine substitutions [12]. We tested 5 HBx mutants containing a substitution of seven consecutive amino acids by the sequence AAASAAA in the region spanning amino acids 30 to 94 (Figure 1C, left panel). HEK293 cells were transfected with HBx mutants and with His-Myc-Spindlin1 vectors and immunoprecipitation assays were carried out using anti-Flag antibodies. We observed that mutants Cm5 and Cm7 containing mutations spanning amino acids 30 to 50 have lost most of their capacity to interact with Spindlin1 (only about 10% and 13% of Spindlin1 are respectively immunoprecipitated) and that mutant Cm13 containing mutations spanning amino acids 88 to 94 interacts weakly with Spindlin1 (29% of Spindlin1 is immunoprecipitated) confirming that these two regions are involved in Spindlin1 interaction (Figure 1C, right panel). Finally, while mutant Cm8 which span amino acids 52 to 58 still interacts with Spindlin1 (52% of Spindlin1 is immunoprecipitated), mutant Cm9 containing mutations spanning amino acids 59 to 65 interacts weakly with Spindlin1 (28% of Spindlin1 is immunoprecipitated) (Figure 1C, right panel). Since the deletion mutant HA-X7 interacts with Spindlin1, these results suggest that alanine substitution in the Cm9 mutant may have induced conformational changes that destabilize Spindlin1 interaction (Figure S1 and Figure 1C, right panel).

The Tudor-like domain II of Spindlin1 has been shown to be important for binding to H3K4me3 and to SERBP1 [24], [26]. To examine whether the binding of Spindlin1 to HBx is dependent on the Tudor domain, we constructed a Spindlin1 mutant containing a deletion of the Tudor-like domain II. HEK293 cells were transfected with His-myc-Spindlin1 wt or His-myc-Spindlin1 deletion mutant (His-Myc-Spin1mut) together with HA-HBx and immunoprecipitations were performed using anti-Myc antibodies. The binding of Spindlin1 deletion mutant to HBx was significantly decreased suggesting that Tudor-like domain II of Spindlin1 is required for HBx interaction (Figure 1D). Altogether, these data confirm that Spindlin1 is a novel cellular partner for HBx.

### Spindlin1 is a repressor factor of HBV transcription

**Figure 2:**
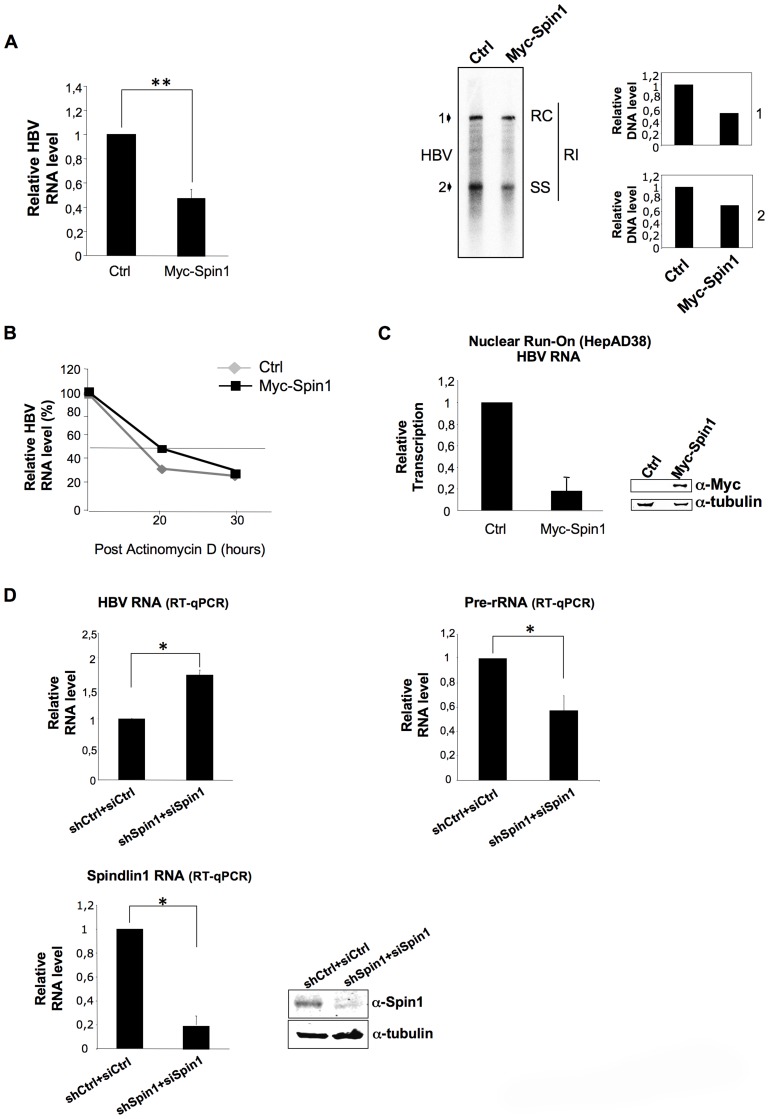
Spindlin1 represses HBV transcription in HepAD38 cells. (**A**) HepAD38 cells cultured without tetracycline were transduced with an empty lentiviral vector (Ctrl) or a lentiviral vector encoding His-Myc-Spindlin1 (Myc-Spin1). Cells were then harvested 48 h after transduction for total RNA extraction and HBV transcription was analyzed by RT-qPCR (left graph). Transcript level in cells transduced with the empty lentiviral vector was set to 1. *P* values were determined by Wilcoxon test (**, *P* = 0.007). Error bars represent SD of eight independent experiments. Alternatively, cells were harvested 72 h after transduction and capsid-associated DNA was extracted. The replication intermediates were then analyzed by southern blot. RC: relaxed circular DNA, SS: single-stranded DNA, RI: replication intermediates. The relative intensities of RC DNA (1) and SS DNA (2) were quantified. The results are presented on the right graphs. The intensity in the control condition was set at 1. (**B**) Spindlin1 does not affect HBV RNA stability. HepAD38 cultured without tetracycline were transduced as in (A). After 48 h, the cells were treated with actinomycin D (5 µg/ml) that blocks polymerase II. Cells were collected at the indicated times after treatment and total RNA was prepared. HBV RNAs were then quantified by RT-qPCR. The relative level of HBV transcription was normalized with Roth2 gene. The amount of RNA at time zero was set at 100%. (**C**) Spindlin1 modulates the rate of HBV transcription. Nuclear run-on assays were performed on isolated nuclei from HepaAD38 cells transduced as in (A). Transcripts generated during run-on were purified using anti-BrdU beads and HBV RNAs were quantified by RT-qPCR. Transcript level in cells transduced with the empty lentiviral vector was set to 1. Error bars represent SD of three independent experiments. The expression of His-Myc-Spin1 was analyzed by immunoblot with anti-Myc antibodies. Tubulin was used as loading control (Right panel). (**D**) Spindlin1 knock-down increases HBV transcription. HepAD38 cells were transduced with a lentiviral vector allowing the expression of a control shRNA (shCtrl) or shRNAs directed against Spindlin1 (shSpindlin1). After selection with puromycin (4 µg/ml), cells stably expressing shCtrl or shSpin1 were transfected with 25 nM of control siRNA (siCtrl) or directed against Spindlin1 (siSpin1) respectively. 48 h post-transfection, cells were harvested for total RNA extraction and, HBV (left panel) or pre-rRNA transcription (right panel) were analyzed by RT-qPCR. Spindlin1 expression in shCtrl+siCtrl or shSpin1+siSpin1 HepAD38 cells was analyzed by RT-qPCR (bottom left graph) and by immunoblotting with anti-Spin1 antibodies. Anti-tubulin immunostaining was used as loading control (bottom right panel). Transcript level in shCtrl HepAD38 cells transfected with siCtrl was set to 1. *P* values were determined by Wilcoxon test (*, *P*<0.05). Error bars represent SD of six independent experiments.

To further determine the functional relevance of HBx and Spindlin1 interaction, we studied the role of Spindlin1 in HBV replication. HepAD38 cells, that contain the HBV genome driven by a minimal cytomegalovirus (CMV) promoter under tetracycline control, were transduced with a lentiviral vector coding for His-Myc-Spindlin1 or with a control lentiviral vector. After 48 h, HBV RNA expression level and HBV replicative intermediates were respectively analyzed by quantitative RT-PCR (qRT-PCR), northern blotting and southern blotting using ^32^P-full-length HBV DNA probe (Figure 2A and Figure S2A). As shown in Figure 2A right panel, the level of capsid associated HBV DNA in cells expressing His-myc Spindlin1 was reduced as compared to control cells. Moreover, the decrease of HBV DNA replicative intermediates in His-myc-Spindlin1 expressing HepAD38 cells is directly correlated with a decrease in HBV RNA level suggesting that Spindlin1 modulates HBV transcription (Figure 2A left panel). To demonstrate the specificity of Spindlin1 activity on HBV transcription, we showed that Spindlin1 overexpression does not modulate CCNA2 (cyclin A2) transcription (Figure S2B). Because in HepAD38 cells HBV RNAs could be transcribed from both integrated DNA and cccDNA, we tested whether Spindlin1 inhibits cccDNA transcription. HepAD38 were treated with tetracycline for 16 hours before transduction with His-Myc-Spindlin1 lentiviral vector or with a control lentiviral vector. Using qRT-PCR, we observed that Spindlin1 overexpression reduced HBV transcription suggesting that Spindlin1 modulates cccDNA transcription (Figure S2C). To next determine whether Spindlin1 modulates HBV RNA stability or rather acts at the level of transcription rate, we examined the HBV RNAs half-life in HepAD38 cells transduced with lentiviral vector coding for His-myc-Spindlin1 or with a control lentiviral vector. Briefly, 48 h after transduction, RNA synthesis was blocked using the RNA polymerase II inhibitor actinomycin D. The level of viral RNAs was determined by RT-qPCR at different time points after actinomycin D addition and normalized by the Roth2 cellular mRNA that was chosen because of its long half-life (unpublished results and Figure 2B). The relative half-life of HBV RNAs does not vary significantly in HepAD38 cells overexpressing or not Spindlin1 suggesting that Spindlin1 does not modulate HBV RNAs stability. We next confirmed that Spindlin1 acts on the rate of HBV transcription using nuclear run-on assays. For this, experiments were performed using nuclei isolated from HepAD38 cells overexpressing or not His-Myc-Spindlin1. Analysis of HBV transcripts generated during the run-on showed that Spindlin1 negatively regulates the transcription of HBV (Figure 2C). The lack of Spindlin1 activity on cyclin A2 transcription confirms that Spindlin1 modulates the transcription of specific target genes (Figure S2D).

Finally, to confirm the role of Spindlin1 on HBV transcription we silenced the expression of Spindlin1 in HepAD38 cells and analyzed HBV transcription using RT-qPCR (Figure 2D). As control we quantified the level of ribosomal RNA (pre-rRNA) known to be positively regulated by Spindlin1 [24]. Depletion of Spindlin1 increased HBV transcription while it decreased pre-rRNA transcription as previously shown (Figure 2D upper left and right graphs). Spindlin1 depletion does not affect cyclin A2 transcription suggesting that Spindlin1 modulates the transcription of a subset of genes (Figure S2E).

Together these results show that Spindlin1 represses HBV replication by acting at the level of transcription.

### Spindlin1 represses HBV transcription in the context of viral infection and its activity is counteracted by HBx

**Figure 3:**
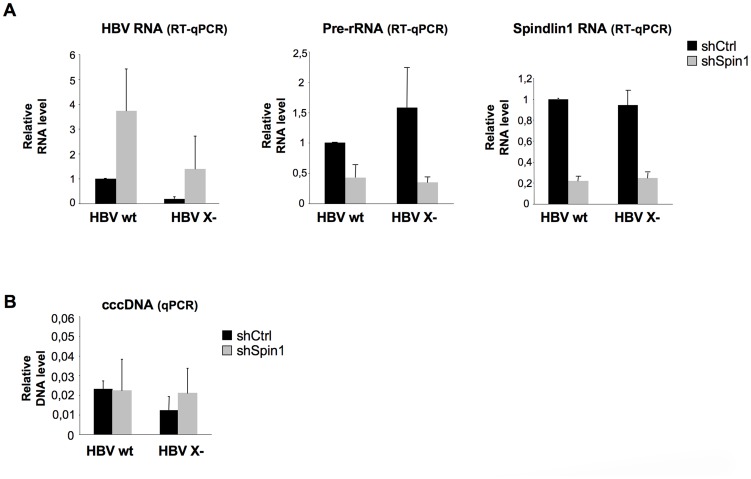
Endogenous Spindlin1 represses HBV transcription in the setting of infection. HepaRG cells stably expressing a shRNA control (shCtrl) or a pool of three shRNA directed against Spindlin1 (shSpin1) were established after transduction with the corresponding lentiviral vectors and selection with puromycin. Infections were then carried out with normalized amount of wild virus (HBV wt) or virus deficient for the expression of HBx (HBV X-). 8 days after infection, cells were harvested for total RNA extraction and cccDNA extraction. (**A**) HBV and pre-rRNA transcription were analyzed by RT-qPCR (left panel and middle panel respectively). Spindlin1 expression in shCtrl or shSpin1 HepaRG cells was analyzed by RT-qPCR (right panel). The relative transcription level was normalized with Roth2 cellular gene. Transcript level in shCtrl HepaRG cells infected with HBV wt was set to 1. Error bars represent SD of three independent experiments. (**B**) Nuclear DNA extraction. DNA was treated with plasmid safe before cccDNA amplification. The amount of cccDNA was quantified by qPCR and normalized to the amount of cyclin A. The cccDNA level in each condition was analyzed by qPCR using primers that amplify the cccDNA. The cccDNA level in shCtrl HepaRG cells infected with HBV wt was set at 1. Error bars represent SD of three independent experiments.

We next studied whether Spindlin1 represses HBV transcription in the context of infection. Moreover, since HBx interacts with Spindlin1, we examined whether Spindlin1 differentially affects the transcription of wild type HBV (HBV wt) and HBx deficient HBV (HBV X-) viruses. HepaRG cells stably expressing the shRNA Spindlin1 (shSpin1) or the shRNA control (shCtrl) were then infected with normalized amount of HBV wt or HBV X- viruses and both HBV and ribosomic transcription were analyzed 8 days later using RT-qPCR. In line with previous report, depletion of Spindlin1 resulted in a decrease of rRNA transcription; in parallel it induced HBV wt and HBV X- transcription (Figure 3A, left and middle graphs). Increase of HBV transcription in Spindlin1-depleted HepaRG cells was accompanied by an increase in HBsAg production in the supernatant (Figure S3A). Interestingly, the increase of HBV RNA level upon Spindlin1 depletion was stronger (7 fold) for the HBV X- virus than for the HBV wt virus (3.4 fold), suggesting that HBx is able to counteract the activity of Spindlin1. To rule out the possibility that Spindlin1 modulates an early step of HBV infection, then modifying the level of cccDNA in the nucleus, we analyzed by qPCR the level of cccDNA in Spindlin1-depleted HepaRG cells or in control cells after infection with HBV wt or HBV X- viruses. As shown in Figure 3B, Spindlin1 knockdown did not modulate cccDNA accumulation. This observation was further confirmed by Southern Blot analysis (Figure S3B). Finally, using a lentiviral vector coding for His-Myc-Spindlin1, we overexpressed Spindlin1 in differentiated HepaRG cells that were infected with normalized amount of HBV wt virus. We observed that overexpression of Spindlin1 repressed the transcription of HBV RNA during infection (Figure S3C).

### Spindlin1 is recruited to the HBV cccDNA and its recruitment is dependent of its Tudor-like domain II

**Figure 4:**
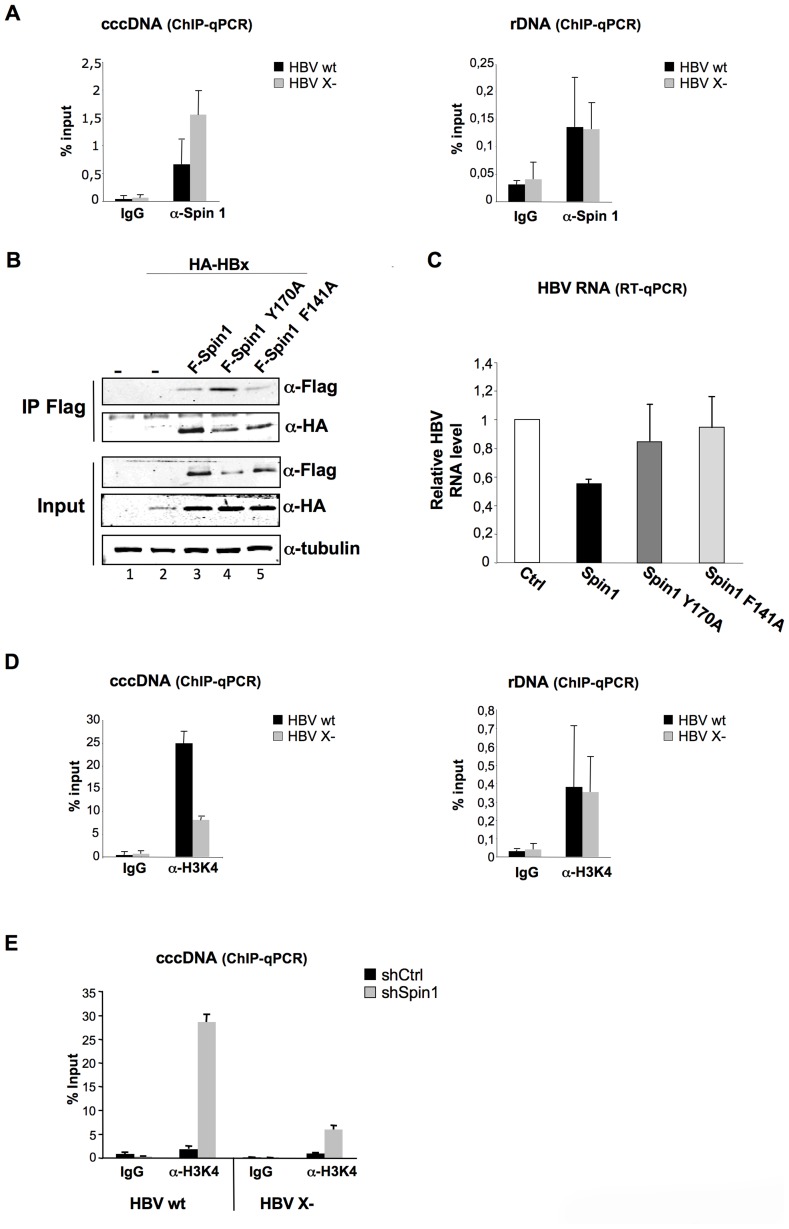
HBx expression correlates with the decrease of Spindlin1 recruitment to the cccDNA. (**A**) Differentiated HepaRG cells were infected with normalized amount of HBV WT or HBV X- viruses. 8 days after infection, cells were harvested and analyzed by ChIP using antibodies against Spin1. As a control, immunoprecipitation was performed with purified rabbit IgG. Input and immunoprecipitated DNA were analyzed in triplicate by qPCR with primers specific for HBV DNA or for rDNA and are displayed as percent input. Error bars represent SD of three independent experiments. (**B**). Spindlin1 mutants deficient for methyllysine binding interact with HBx. HEK293 cells were transfected with a plasmid coding for HA-HBx protein alone or in combination with plasmids coding for wild type Flag-Spindlin1 protein (F-Spin1) or for the Flag-tagged Spindlin1 mutants Y170A and F141A (F-Spin1 Y170A and F-Spin1 F141A respectively). After anti-Flag immunoprecipitation, proteins were analyzed by anti-HA and anti-Flag immunoblotting. Tubulin was used as a loading control. (**C**) Repression of cccDNA transcription by Spindlin1 is dependent of its ability to interact with methyllysine. HepAD38 cells were transfected by electroporation with plasmids encoding F-Spin1, F-Spin1 Y170A or F-Spin1 F141A. 48 h after transfection, cells were harvested and total RNA was extracted. HBV transcription was evaluated by RT-qPCR. Transcript level in cells transfected with the Ctrl plasmid was set to 1. Error bars represent SD of four independent experiments. (**D**) The level of H3K4me3 associated to the cccDNA or to the rDNA was studied by ChIP-qPCR with anti-H3K4me3 antibodies. As a control, immunoprecipitation was performed with purified rabbit IgG. Input and immunoprecipitated DNA were analyzed in triplicate by qPCR with primers specific for HBV DNA or for rDNA and are displayed as percent input. Error bars represent SD of three independent experiments. (**E**) Differentiated shCtrl or shSpin1 HepaRG cells were infected with normalized amount of HBV WT or HBV X- virus. 8 days after infection, cells were harvested and analyzed by ChIP assay using antibodies against H3K4me3. As a control, immunoprecipitation was performed with purified rabbit IgG. Input and immunoprecipitated DNA were analyzed in triplicate by qPCR with primers specific for HBV DNA and are displayed as percent input. Error bars represent SD of three independent experiments.

To investigate the role of Spindlin1 on HBV transcription, we studied its recruitment to the cccDNA. Eight days after infection of differentiated HepaRG cells with normalized amount of HBV wt or HBV X- viruses, chromatin was prepared and subjected to quantitative Chromatin immunoprecipitation (ChIP-qPCR) assays using the indicated antibodies. As shown in Figure 4A, Spindlin1 was recruited to the HBV cccDNA and importantly, its recruitment to the HBV X- cccDNA was increased compared to the HBV wt cccDNA (Figure 4A left panel). Consistent with previous reports we observed the recruitment of Spindlin1 to the ribosomal DNA (Figure 4A right panel).

We then studied whether the aromatic cage of the Tudor-like domain II of Spindlin1 needed for methyllysine binding is required for the transcriptional repression of HBV. To this aim we tested the activity of previously described Spindlin1 constructs containing mutation in their Tudor-like domain II. Mutation at F141 residue (Spindlin1 F141A) and mutation at residue Y170 (Spindlin1 Y170A) both lead to a decrease in H3K4me3 binding and in the activation of pre-rRNA transcription [20], [24]. We first studied whether these two mutants are able to interact with HBx. HEK293 cells were transfected with Flag-Spindlin1 wild type or Flag-Spindlin1 mutants (Y170A and F141A) and HA-HBx vectors and immunoprecipitation assays were performed using anti-Flag antibodies. Wild-type and mutants Spindlin1 proteins coimmunoprecipate with HA-HBx (Figure 4B). HBV RNA transcription was next assessed in HepAD38 cells electroporated with vectors coding Flag-Spindlin1 wild type or Flag-Spindlin1 Y170A or F141A mutants. As shown in Figure 4C, Spindlin1 Y170A and F141A mutants, in contrast with wt Spindlin1, did not affect HBV transcription suggesting that transcriptional repression by Spindlin1 is dependent on its Tudor-like domain II.

A previous study has shown that the recruitment of Spindlin1 to the rDNA correlates with the increase of H3K4me3 marks [24]. ChIP experiments performed with anti-H3K4me3 antibodies and chromatin prepared from HepaRG cells infected with normalized amount of HBV wt or HBV X- viruses, showed that wt cccDNA is preferentially associated with H3K4me3 marks compared to the X- cccDNA (Figure 4D). Our results suggest that in the case of HBV cccDNA there is an inverse correlation between the recruitment of Spindlin1 and H3K4me3. We next studied whether depletion of Spindlin1, which is associated with increased HBV transcription, correlates with the deposition of active marks (H3K4me3). ChIP-qPCR assays were performed using chromatin prepared from Spindlin1-depleted HepaRG cells or control HepaRG cells infected with normalized amount of HBV wt or HBV X- viruses. Depletion of Spindlin1 leads to a significant increase in H3K4me3 (Figure 4E).

Together our data suggest that cccDNA transcriptional repression by Spindlin1 correlates with its recruitment to the cccDNA and with the decrease of H3K4me3; a process that required Tudor-like domain II integrity.

### Spindlin1 represses the transcription of HSV-1

**Figure 5:**
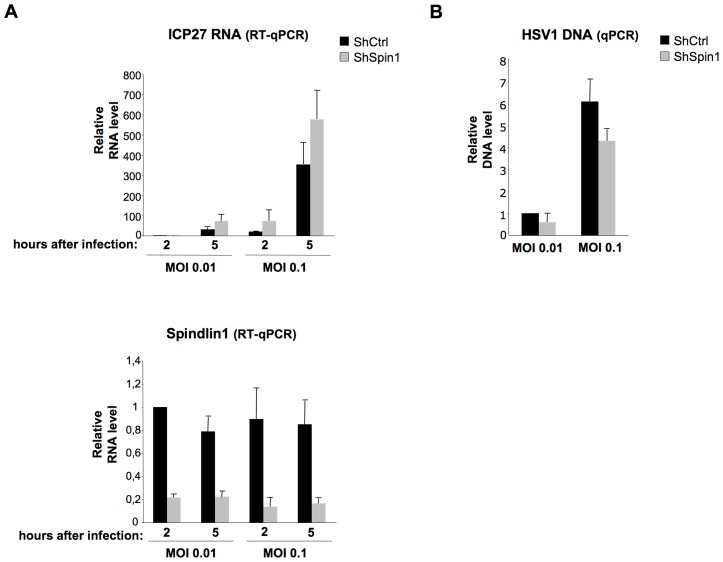
Spindlin1 represses the transcription of HSV-1 during infection. ShCtrl or shSpin1 HepaRG cells were infected in duplicate with HSV1 at MOI 0.01 and 0.1. Cells were harvested at 2 h and 5 h post-infection for total RNA extraction and viral DNA extraction. (**A**) HSV-1 ICP27 immediate early RNA transcription was quantified by RT-qPCR (upper panel). Spindlin1 expression in shCtrl or shSpin1 HepaRG cells was analyzed by RT-qPCR (bottom panel). Transcript level in shCtrl HepaRG cells 2 h after HSV-1 infection was set to 1. Error bars represent SD of two independent experiments. (**B**)The amount of HSV-1 DNA in shCtrl HepaRG cells 2 h after infection was analyzed by qPCR. HSV-1 DNA level in shCtrl HepaRG cells 2 h after HSV-1 after infection was set to 1. Error bars represent SD of two independent experiments.

Having shown that Spindlin1 represses the transcription of HBV, we asked whether it could also repress the transcription of different DNA viruses that replicate in the nucleus. To this aim, we tested the role of Spindlin1 on the transcription of Herpes simplex virus type 1 (HSV-1). Upon HSV-1 infection, the viral nucleocapsid is transported to the nuclear pores and the viral genome is released into the nucleus. HSV-1 transcription that takes place after the circularization of the linear viral DNA is temporally organized with transcription of immediate early (IE), early and late gene products [34]. In order to investigate the role of Spindlin1 on HSV-1 transcription, shCtrl or shSpin1 HepaRG cells were infected with normalized amount of HSV-1 using two different MOI (0.01 and 0.1). HSV-1 transcription was analyzed 2 h and 5 h post-infection by RT-qPCR to detect ICP27 transcripts, which can be conveniently used as a marker of transcription, as previously shown [35]. Depletion of Spindlin1 increased HSV-1 transcription at both MOI (Figure 5A). We confirmed that input DNA was similar in infected cells using qPCR that amplifies HSV-1 DNA (Figure 5B).

To study whether this negative regulation is specific to DNA viruses, we tested the role of Spindlin1 overexpression on the replication of the Hepatitis C virus (HCV), a positive strand RNA virus. Huh7.25.CD81 cells were first transfected with increasing amount of His-Myc-Spindlin1 vector and 24 h later, the cells were infected with normalized amount of HCV at MOI 0.3. HCV replication was assessed by RT-qPCR 48 h after infection. As shown in Figure S4, Spindlin1 overexpression did not modulate HCV replication.

All together our data suggest that Spindlin1 is involved in the negative regulation of the transcription of the DNA viruses in the nucleus.

## Discussion

A major finding in this manuscript is that Spindlin1, a protein that we isolated as an HBx interacting partner, represses the transcription of not only HBV but also HSV-1 through epigenetic regulation, suggesting that Spindlin1 could participate in the recognition and the silencing of these two viral nuclear DNAs. Indeed, we observed that suppression or overexpression of Spindlin1 respectively increases or inhibits the transcription of both HBV and HSV-1 in the context of HepaRG cells infection. Using nuclear run-on assays, we showed that Spindlin1 acts at the level of transcription. Finally, using ChIP experiments, we demonstrated that Spindlin1 inhibits transcription through its recruitment to the viral DNA and that its depletion correlates with an increase of active chromatin marks. Importantly, we observed that HBV transcriptional repression by Spindlin1 is higher for the HBV X- virus than for the HBV wt virus, suggesting that HBx via its interaction with Spindlin1 counteracts its activity. This hypothesis is further supported by the fact that Spindlin1 is recruited at a higher level to the HBV X- cccDNA than to the HBV wt cccDNA.

Previous studies have shown that HBx is essential for the establishment of productive infection by acting mainly at the level of transcription. Indeed Lucifora and colleagues have observed that HBV X - infection leads to a very low level of HBV RNA that correlates with the decrease of H3 acetylation onto the cccDNA [13]. Their findings suggest that HBx might control HBV transcription by favoring the establishment of a transcriptionally active chromatin structure. This is in agreement with data showing that the recruitment of HBx to the cccDNA, reconstituted after transfection of HepG2 cells with a linear HBV genome, correlates with the hyperacetylation of histone H3 and H4 and with HBV transcription [6], [10]. Finally, in a similar model, Colgrove and colleagues demonstrated using nuclear run-on that HBx acts by increasing the rate of HBV RNA transcription [11]. Increasing evidence from the literature suggests that while HBx could activate transcription through the recruitment of coactivators favoring hyperacetylation and transcription, it might in first line counteract the activity of repressor(s) that silence virus transcription. This hypothesis is further supported by the work from Van Breugel and colleagues who showed that HBx increases the transcription of any genes located onto episomic DNA regardless of their promoter or enhancer [15]. Here we have isolated by tandem affinity purification a cellular protein Spindlin1 that inhibits the transcription of two different DNA viruses that remain as episomal DNA into the nucleus. Spindlin1 is recruited directly to the cccDNA and using run-on assay or by blocking RNA transcription with actinomycin D, we could demonstrate that Spindlin1 blocks directly HBV transcription. Finally, we have demonstrated that silencing of Spindlin1 restores HBV X- transcription, which correlates with increased H3K4me3. Thus by counteracting Spindlin1, HBx may favor the establishment of active chromatin on the cccDNA, which is in accordance with previous report showing that expression of HBx leads to histone hyperacetylation and HBV transcription [10], [13]. Together our findings demonstrate that Spindlin1 is one of the factors involved in the silencing of HBV cccDNA. Further work will be needed however to determine whether Spindlin1 represses only episomal DNA or also integrated DNA.

Transcriptional activation of rRNA by Spindlin1 requires its interaction with H3K4me3 [24]. However, the mechanism involved in this activation is unknown. Recently, Spindlin1 has been shown to promote Wnt/β-catenin signaling. An interaction between Spindlin1 and the β-catenin partner Tcf4 has been demonstrated, but whether it is required for Wnt/β-catenin activation and whether this interaction involved the methylation of Tcf4 is unknown [36]. A recent publication showed that Spindlin1 is able to simultaneously recognize H3K4me3 and H3 asymetric dimethylarginine 8 (H3R8me2a) through Tudor-like domain II and I respectively [25]. The study further suggests that Spindlin1, as a histone reader, senses both H3K4me3 and H3R8me2a on Wnt/β-catenin target genes and activates transcription [25]. Finally, the Tudor-like domain II was recently shown to be required for the interaction between Spindlin1 and SERBP1, but again whether SERBP1 methylation is involved is unknown [26]. In our study we observed that Spindlin1 recruitment to the cccDNA is inversely correlated with H3K4me3, suggesting that it is independent of this activating mark. Nevertheless, we observed that the amino acid residues in the Tudor-like domain II that are critical for the binding of Spindlin1 to H3K4me3 and for the formation of the aromatic cage needed for methyllysine binding are also critical for HBV repression. Our results suggest that while H3K4me3 is not involved in Spindlin1 recruitment, an as yet unidentified methylated protein might be responsible for its recruitment. Of note, a direct interaction of Spindlin1 with dsDNA has also been described. The authors demonstrated that Spindlin1 has higher affinity for super helical than for open cycle dsDNA [21]. While tudor-like domains can be involved in nucleic acid binding, the domain responsible for the binding of Spindlin1 to dsDNA has not yet been identified. HBV and HSV-1 DNA enter into the nucleus as naked DNA. Could Spindlin1 be responsible for the early recognition of viral DNA? Further study will be needed to determine whether Spindlin1 recognizes only viral DNA or both the DNA and a methylated protein in order to mediate the establishment of repressive chromatin.

Spindlin1 has been to date identified in different complexes such as the β-arrestin complex, Argonaute 3 complex and to interact with different proteins such as H3K4me3, Tcf4, SERBP1 and hyaluronan binding protein 4 (HABP4), suggesting that Spindlin1 is involved in diverse cellular processes [24], [26], [36], [37], [38], [39]. In oocyte, Spindlin1 interacts and cooperates with SERBP1 to regulate mRNA stability [26]. Our data show that Spindlin1 does not modulate HBV RNA stability but acts mainly at the level of HBV transcription. Interestingly, SERBP1 and HABP4, identified as Spindlin1 interacting partners using a two hybrid screen, interact with the chromo-helicase-DNA-binding protein3 (CHD3) [40]. CHD3 possesses ATPase activity and is part of the nucleosome remodeling and deacetylase (NuRD) complex that regulates chromatin structure and gene transcription [41]. The identification of Spindlin1 interacting partners will thus help understanding how Spindlin1 represses viral transcription and whether it acts via its interaction with repressor factors by assembling repressive chromatin at the viral DNA.

While to date, Spindlin1 has been identified as a transcriptional activator, we observed here that Spindlin1 represses the transcription of HBV and HSV-1 in the context of infection. The molecular mechanisms of both transcriptional activation and transcriptional repression by Spindlin1 is yet unknown. The switch from a repressor function to that of an activator can be dependent on the partner associated with Spindlin1 and/or on post-translational modifications. It has been shown that lysine-specific demethylase 1 (LSD1) behave as a repressor through the demethylation of H3K4me1/2 when associated with CO-REST a chromatin-associated transcriptional repressor but as an activator when associated with androgen receptor through the demethylation of H3K9 [42], [43]. Recently the dual role of LSD1 has been demonstrated in Notch-1 signaling pathway. LSD1 functions as a corepressor of Notch1 target genes when associated with CSL-repressor complex. Upon Notch1 activation, LSD1 is part of Notch-activation complex and contributes to gene activation through H3K9me2 demethylation [44]. The Jak/STAT signaling pathway is known to mediate gene activation through the phosphorylation of the signal transducer and activator of transcription (STAT) that translocates into the nucleus and stimulates transcription of target genes. However it has been shown that, at least in drosophila, unphosphorylated STAT associates with the Drosophila homolog of heterochromatin1 (HP1) Su(var)2–5 and maintains the stability of transcriptionally repressed chromatin [45].

We also showed that Spindlin1 represses the transcription of HSV-1, as its depletion in HepaRG increases the transcription of early ICP27 transcripts used as a marker of viral transcription. Upon entry into the nucleus, HSV-1 gene expression is rapidly repressed by constitutively expressed proteins such as components of the PML nuclear body : hDaxx, ATRX, Sp100 and PML, as well as proteins of the DNA repair machinery such as RNF8, RNF168 and 53BP1 [46]. The chicken homolog of Spindlin1 (chSpin-W) has been shown to colocalize with SUMO-1 in nuclear dots in interphase cells, suggesting that chSpin-W localizes to PML nuclear bodies [47]. It will thus be interesting to study whether Spindlin1 also localizes to PML bodies in cells in normal condition or during infection. Recruitment of PML components or DNA repair proteins to the incoming viral DNA involves many different post-translational modifications such as sumoylation and ubiquitination [48], [49]. While the importance of protein methylation in these processes has not been studied to our knowledge, methylation is involved in the recruitment of DNA repair complexes to sites of DNA damage. For example, it has been shown that H3K79me2 mediates the recruitment of 53BP1 to DNA double-strand breaks and methylation of 53BP1 or Mre11 modulates both their activities and their recruitment to DNA damage sites [50], [51], [52]. Further studies will be needed to determine how Spindlin1 represses HSV-1 transcription and whether this repression is similar to HBV repression and requires the Tudor like-domain II.

Spindlin1 was identified as a partner of HBx. Using HBx deletion mutants and HBx alanine mutants; we observed that the N-terminal domain of HBx to aa 50, as well as domains spanning aa 59 to 65 and aa 84 to 140 are needed for a maximal interaction with Spindlin1. A former study using HBx alanine mutants has shown that amino acids 52 to 65 and 88 to 154 are critical for the transcriptional activation and the replication of an HBV X- vector transfected into HepG2 cells [12]. The inability of HBx proteins containing mutation in the domains spanning aa 59 to 65 and aa 88 to 154 to restore HBV transcription/replication can be in part be linked to their weak interaction with Spindlin1. Further study will be needed to assess if an HBx mutant deficient for Spindlin1 interaction is fully or only partially competent to restore HBV transcription in the setting of infection. Our observation that in the setting of infection less Spindlin1 is recruited to the wt HBV cccDNA than to the HBV X- cccDNA suggests that HBx is the viral factor that counteracts the repressive activity of Spindlin1. This is in line with our observation that the HBV X- virus is more strongly repressed by Spindlin1. Different hypotheses could be put forward to explain how HBx counteracts Spindlin1 activity. Preliminary data shows that the Tudor-like domain II of Spindlin1 is required for the interaction with HBx. It is thus possible that by binding to Spindlin1, HBx delocalizes Splindin1 away from the cccDNA. HBx has been shown to interact with the E3 ubiquitin ligase Cul4A/DDB1, which is essential for both HBV replication and HBx transcriptional activity [53], [54]. In order to counteract cellular restriction factors and favor their replication, viruses either encode for E3 ubiquitin ligase or hijack cellular E3 ubiquitin ligase. For example HSV-1 codes for the ICP0 protein that possesses ubiquitin ligase activity and induces the degradation of restriction factors such as the ATRX, RNF8 and RNF168 and PML proteins [46]. Human immunodeficiency virus (HIV-1) codes for the viral protein Vif that interacts with the E3 ubiquitin ligase Cul5/elongin B and C complex and induces the degradation of the restriction factor APOBEC3G [55]. It is thus possible that HBx counteracts Spindlin1 through its binding to the E3 ubiquitin ligase Cul4A/DDB1. We however did not observe the degradation of Spindlin1 in cells overexpressing HBx (data not shown). Alternatively, HBx may modulate Spindlin1 localization or activity through its ubiquitination or could induce the degradation of a Spindlin1 partner.

Owing to the development of cell lines that support infection such as HepaRG, it is now possible to assess the function of cellular and viral proteins on HBV replication in the setting of infection. Here we have identified Spindlin1 as a cellular factor that represses the transcription of DNA viruses such as HSV-1 and HBV. Taken together our results suggest that Spindlin1 contributes to the intrinsic antiviral defense against at least two DNA viruses and that in the case of HBV, its repressive activity is counteracted by HBx.

## Supporting Information

Figure S1 Determination of the region of HBx interacting with Spindlin1. Left panel, schematic representation of full-length HBx protein and in frame HBx deletion mutants. Right panel: HEK293 cells were co-transfected with wild type HA-tagged HBx (HA-X0) construct or HA-tagged HBx deletion mutants (HA-X5, HA-X6, HA-X7, HA-X9 and HA-X10) and the His-myc-Spin1 plasmid. Cellular extracts were immunoprecipitated with anti-HA antibodies and analyzed by anti-Myc and anti-HA immunoblot. The expression of tubulin was used as loading control.
(TIF)

Figure S2 (**A**) HepAD38 cells cultured without tetracycline were transduced with an empty lentiviral vector or a lentiviral vector encoding His-myc-Spindlin1 (Myc-Spin1). 48 h after transduction cells were harvested for total RNA extraction. Transcription was analyzed by Northern blotting. (**B**) HepAD38 cells cultured and transduced as in (A) were harvested 48 h after transduction for total RNA extraction and Cyclin A2 transcription was analyzed by RT-qPCR. Transcript level in cells transduced with the empty lentiviral vector was set to 1. Error bars represent SD of three independent experiments. (**C**) HepAD38 cells grown without tetracycline for several days, were treated with tetracycline for 16 h before transduction with an empty lentiviral vector or a lentiviral vector encoding His-myc-Spindlin1 (Myc-Spin1). Total RNA were prepared as in (B) and HBV transcription was analyzed by RT-qPCR. Error bars represent SD of two independent experiments. (**D**) HepaAD38 cells were transduced as in (B) and nuclear run-on assays were performed on isolated nuclei. Transcripts generated during run-on were purified using anti-BrdU beads and Cyclin A2 RNAs were quantified by RT-qPCR. Transcript level in cells transduced with the empty lentiviral vector was set to 1. Error bars represent SD of three independent experiments. (**E**) HepAD38 shCtrl cells or HepAD38 shSpindlin1 were transfected with 25 nM of control siRNA (siCtrl) or directed against Spindlin1 (siSpin1) respectively. 48 h post-transfection, cells were harvested for total RNA extraction and Cyclin A2 transcription was analyzed by RT-qPCR. Transcript level in HepAD38 shCtrl+ siCtrl cells was set to 1. Error bars represent SD of four independent experiments.
(TIF)

Figure S3 (**A**) Culture supernatants of shSpin1 or shCtrl HepaRG cells were collected 8 days after infection with normalized amount of HBV wt or HBV X- viruses. Secreted HBsAg was measured by ELISA. Secreted HBsAg level in shCtrl cells infected with HBV wt was set at 1. (**B**) Differentiated shSpin1 or shCtrl HepaRG cells were infected with normalized amount of HBV wt HBV X- viruses at MOI 1000. 8 days after infection, cells were harvested for total DNA extraction. Viral DNA was analyzed by Southern blot hybridization using ^32^P labeled HBV-DNA probe. 30 µg of total DNA extracted from HepaD38 cells were used as control (**C**) Differentiated HepaRG cells were transduced with an empty lentiviral vector (Ctrl) or a lentiviral vector encoding His-myc-Spindlin1 (Myc-Spin1). 24 h post-transduction, cells were infected at MOI 200 with HBV wt virus. 8 days after infection, cells were harvested and total RNA was isolated. Viral RNAs were analyzed by RT-qPCR. The level of transcription in the cells transduced with the control lentiviral vector was set at 1. The expression of His-myc-Spindlin1 was analyzed by anti-Myc immunoblot (*: none specific band).
(TIF)

Figure S4 Huh7.25.CD81 cells transfected with 1 or 2 µg of plasmid coding for His-myc-Spindlin1 or with a control plasmid, were infected with HCV at MOI 0.3. 48 h after infection, cells were collected for RNA extraction. Viral RNA was quantified by RT-qPCR. Spindlin1 expression was analyzed by immunoblotting with anti-Myc antibodies.
(TIF)
