# Correction: The Tudor Domain Protein Spindlin1 Is Involved in Intrinsic Antiviral Defense against Incoming Hepatitis B Virus and Herpes Simplex Virus Type 1

**DOI:** 10.1371/journal.ppat.1009135

**Published:** 2020-12-11

**Authors:** Aurélie Ducroux, Shirine Benhenda, Lise Rivière, O. John Semmes, Monsef Benkirane, Christine Neuveut

Following the publication of this article [1], concerns were raised with the presence of irregularities in the background of the western blot results presented in Fig 1D and Fig 2C. The authors indicated that the gels presented in Fig 1D were spliced during figure preparation to show pertinent lanes only. The western blot results presented in Supporting Information file S2 below demonstrate the original results prior to gel splicing. The figure legend of Fig 1 has been updated to clarify that the gels presented in Fig 1D have been spliced. In addition, the authors clarified that the irregularity in the background of Fig 2C is likely the result of an image artifact introduced by the scanning of the blot. The original blot underlying the Fig 2C results, presented in Supporting Information file S4, supports the published figure.

**Fig 1:**
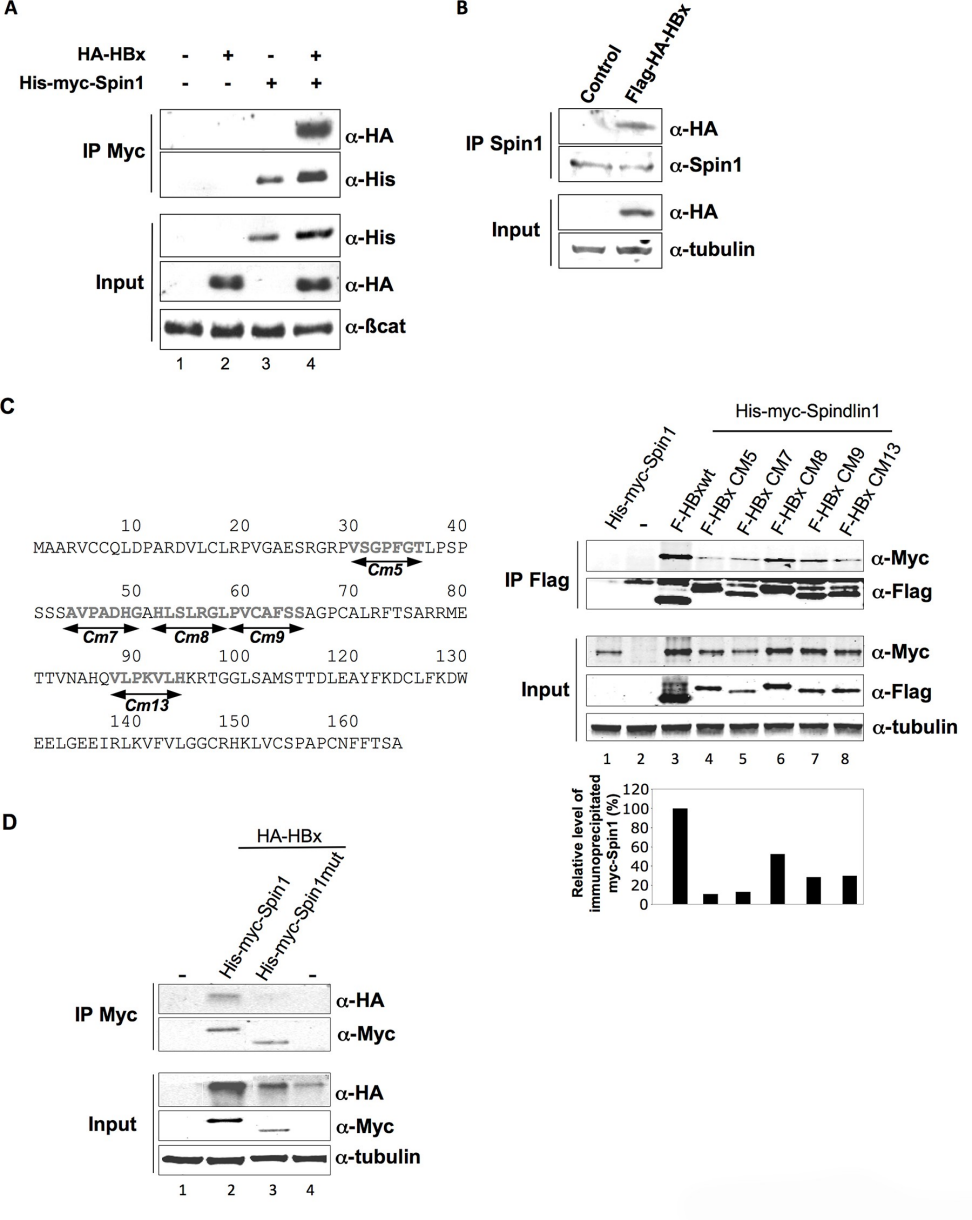
Spindlin1 interacts with HBx. (**A**) Coimmunoprecipitation of His-myc-Spindlin1 (His-myc-Spin1) with HA-HBx using anti-Myc antibodies in HEK293 cells. Proteins in the immune complexes were revealed by Western blotting with anti-His and anti-HA antibodies. The expression of β-catenin was used as loading control. (**B**) Whole-cell extracts, prepared from HepG2 cells transduced with a lentiviral vector encoding Flag-HA-HBx, were immunoprecipitated with anti-Spindlin1 antibodies (IP Spin1). Proteins were detected by Western blotting using anti-HA or anti-Spin1 antibodies. The expression of tubulin was used as loading control. (**C**) Schematic representation of the HBx clustered alanine substitution mutants (left panel). HEK293 cells were co-transfected with Flag-tagged HBx wt construct or the HBx alanine substitution mutants (F-HBx Cm5, F-HBx Cm7, F-HBx Cm8, F-HBx Cm9, F-HBx Cm13) and the His-myc-Spin1 plasmid. Cell extracts were immunoprecipitated with anti-Flag antibodies and analyzed by anti-Myc and anti-Flag immunoblot using the Odyssey system (right panel). The expression of tubulin was used as loading control. Signal strengths of the co-immunoprecipitated His-myc-Spindlin1 proteins were normalized to the ratio of HBx in the IP/HBx in the input. Immunoprecipitated His-myc-Spindlin1 level in cells expressing His-myc-Spindlin1 and Flag-HBx wt was set to 100% (lower right graph) (**D**) Extracts from HEK293 cells transfected with HA-HBx in combination with His-Myc-Spin1 or His-myc-Spindlin1 mutant containing a deletion of the Tudor-like domain II (His-myc-Spin1 mut) were immunoprecipitated with anti-Myc antibodies. Proteins were detected by Western blotting using anti-HA or anti-Myc antibodies. The expression of tubulin was used as loading control. The blots presented in Fig 1D have been spliced in order to present pertinent results only. The full, uncropped blots underlying these results have been provided as Supplementary Information files.

The original blots underlying the results presented in Figs 1C and 1D, 2A 2C and 2D and 4B are presented in the Supporting Information files S1-S5. The authors have not clarified the availability of the underlying data for other results in the article. Interested readers may contact the corresponding author.

## Supporting information

S1 File Original blots underlying Fig 1C. (TIF)

S2 File Original blots underlying Fig 1D. (TIF)

S3 File Original blot underlying Fig 2A. (TIF)

S4 File Original blots underlying Fig 2C and 2D. (TIF)

S5 File Original blots underlying Fig 4B. (TIF)
