# Supplementary figures and images for: The Tudor Domain Protein Spindlin1 Is Involved in Intrinsic Antiviral Defense against Incoming Hepatitis B Virus and Herpes Simplex Virus Type 1
Source: PLoS Pathog. 2014 Sep 11;10(9):e1004343. doi: 10.1371/journal.ppat.1004343 (PMC4161474; doi:10.1371/journal.ppat.1004343)

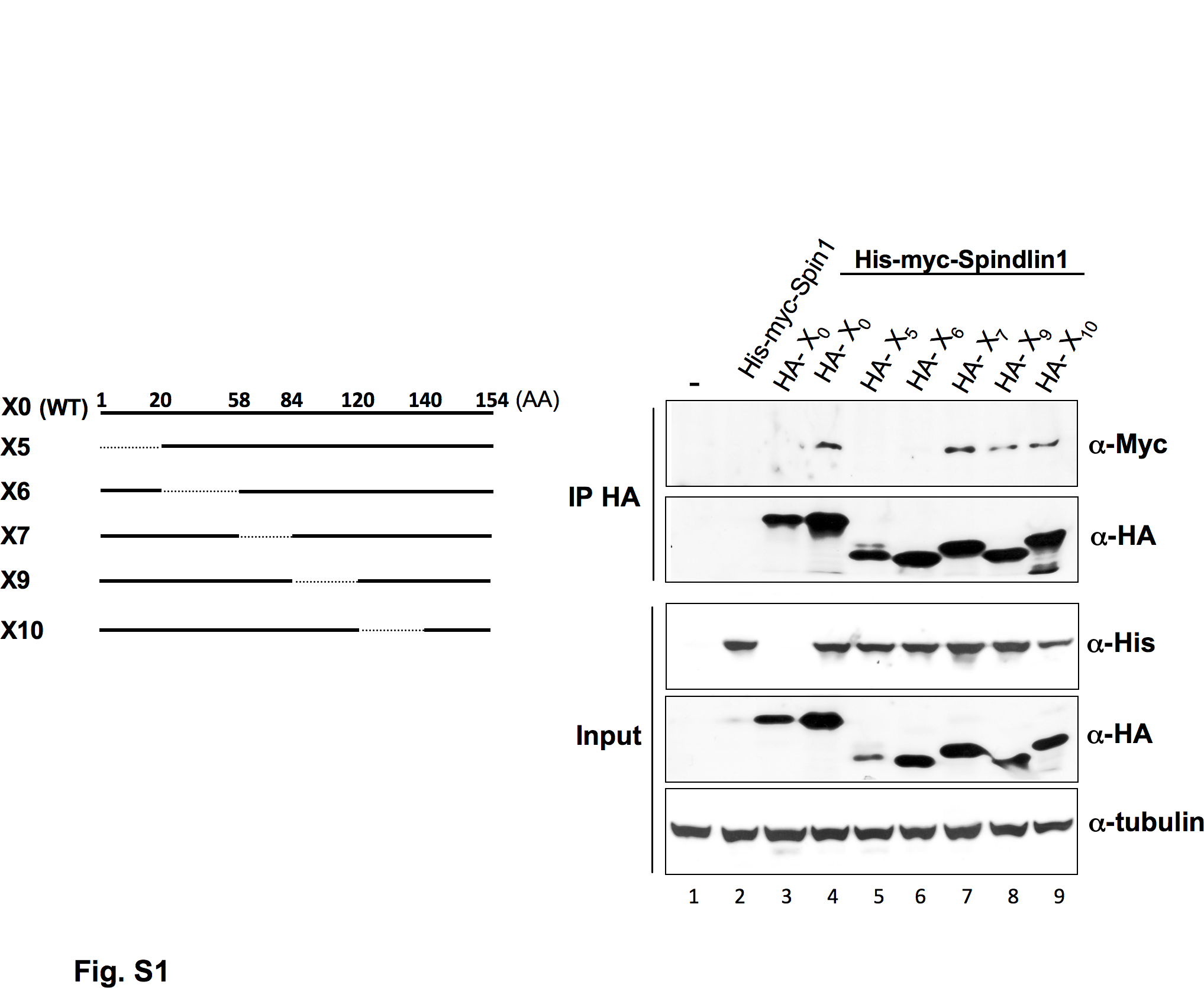

Supplement: Figure S1 — Determination of the region of HBx interacting with Spindlin1. Left panel, schematic representation of full-length HBx protein and in frame HBx deletion mutants. Right panel: HEK293 cells were co-transfected with wild type HA-tagged HBx (HA-X0) construct or HA-tagged HBx deletion mutants (HA-X5, HA-X6, HA-X7, HA-X9 and HA-X10) and the His-myc-Spin1 plasmid. Cellular extracts were immunoprecipitated with anti-HA antibodies and analyzed by anti-Myc and anti-HA immunoblot. The expression of tubulin was used as loading control. (TIF) [file ppat.1004343.s001.tif]

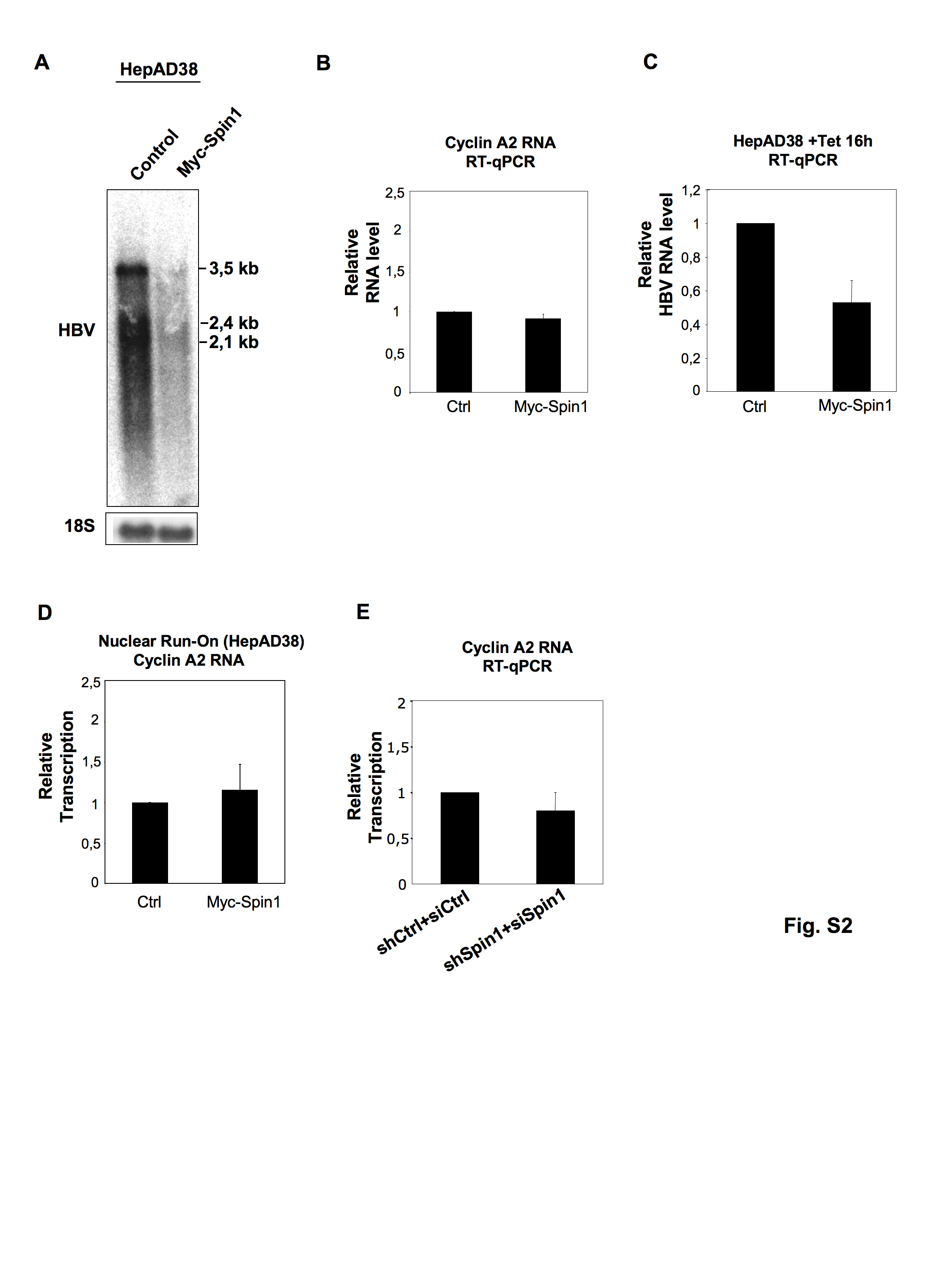

Supplement: Figure S2 — (A) HepAD38 cells cultured without tetracycline were transduced with an empty lentiviral vector or a lentiviral vector encoding His-myc-Spindlin1 (Myc-Spin1). 48 h after transduction cells were harvested for total RNA extraction. Transcription was analyzed by Northern blotting. (B) HepAD38 cells cultured and transduced as in (A) were harvested 48 h after transduction for total RNA extraction and Cyclin A2 transcription was analyzed by RT-qPCR. Transcript level in cells transduced with the empty lentiviral vector was set to 1. Error bars represent SD of three independent experiments. (C) HepAD38 cells grown without tetracycline for several days, were treated with tetracycline for 16 h before transduction with an empty lentiviral vector or a lentiviral vector encoding His-myc-Spindlin1 (Myc-Spin1). Total RNA were prepared as in (B) and HBV transcription was analyzed by RT-qPCR. Error bars represent SD of two independent experiments. (D) HepaAD38 cells were transduced as in (B) and nuclear run-on assays were performed on isolated nuclei. Transcripts generated during run-on were purified using anti-BrdU beads and Cyclin A2 RNAs were quantified by RT-qPCR. Transcript level in cells transduced with the empty lentiviral vector was set to 1. Error bars represent SD of three independent experiments. (E) HepAD38 shCtrl cells or HepAD38 shSpindlin1 were transfected with 25 nM of control siRNA (siCtrl) or directed against Spindlin1 (siSpin1) respectively. 48 h post-transfection, cells were harvested for total RNA extraction and Cyclin A2 transcription was analyzed by RT-qPCR. Transcript level in HepAD38 shCtrl+ siCtrl cells was set to 1. Error bars represent SD of four independent experiments. (TIF) [file ppat.1004343.s002.tif]

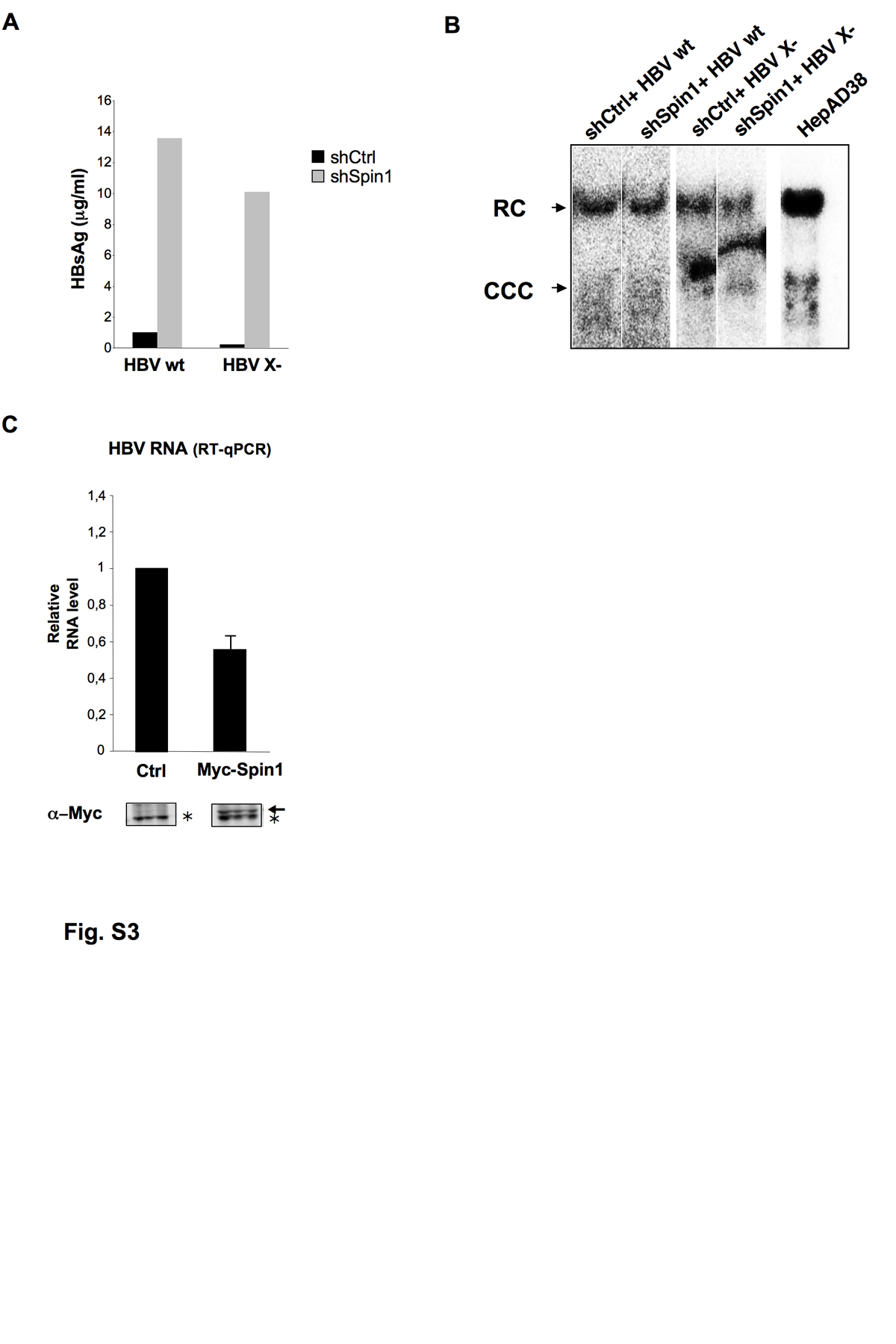

Supplement: Figure S3 — (A) Culture supernatants of shSpin1 or shCtrl HepaRG cells were collected 8 days after infection with normalized amount of HBV wt or HBV X- viruses. Secreted HBsAg was measured by ELISA. Secreted HBsAg level in shCtrl cells infected with HBV wt was set at 1. (B) Differentiated shSpin1 or shCtrl HepaRG cells were infected with normalized amount of HBV wt HBV X- viruses at MOI 1000. 8 days after infection, cells were harvested for total DNA extraction. Viral DNA was analyzed by Southern blot hybridization using 32P labeled HBV-DNA probe. 30 µg of total DNA extracted from HepaD38 cells were used as control (C) Differentiated HepaRG cells were transduced with an empty lentiviral vector (Ctrl) or a lentiviral vector encoding His-myc-Spindlin1 (Myc-Spin1). 24 h post-transduction, cells were infected at MOI 200 with HBV wt virus. 8 days after infection, cells were harvested and total RNA was isolated. Viral RNAs were analyzed by RT-qPCR. The level of transcription in the cells transduced with the control lentiviral vector was set at 1. The expression of His-myc-Spindlin1 was analyzed by anti-Myc immunoblot (*: none specific band). (TIF) [file ppat.1004343.s003.tif]

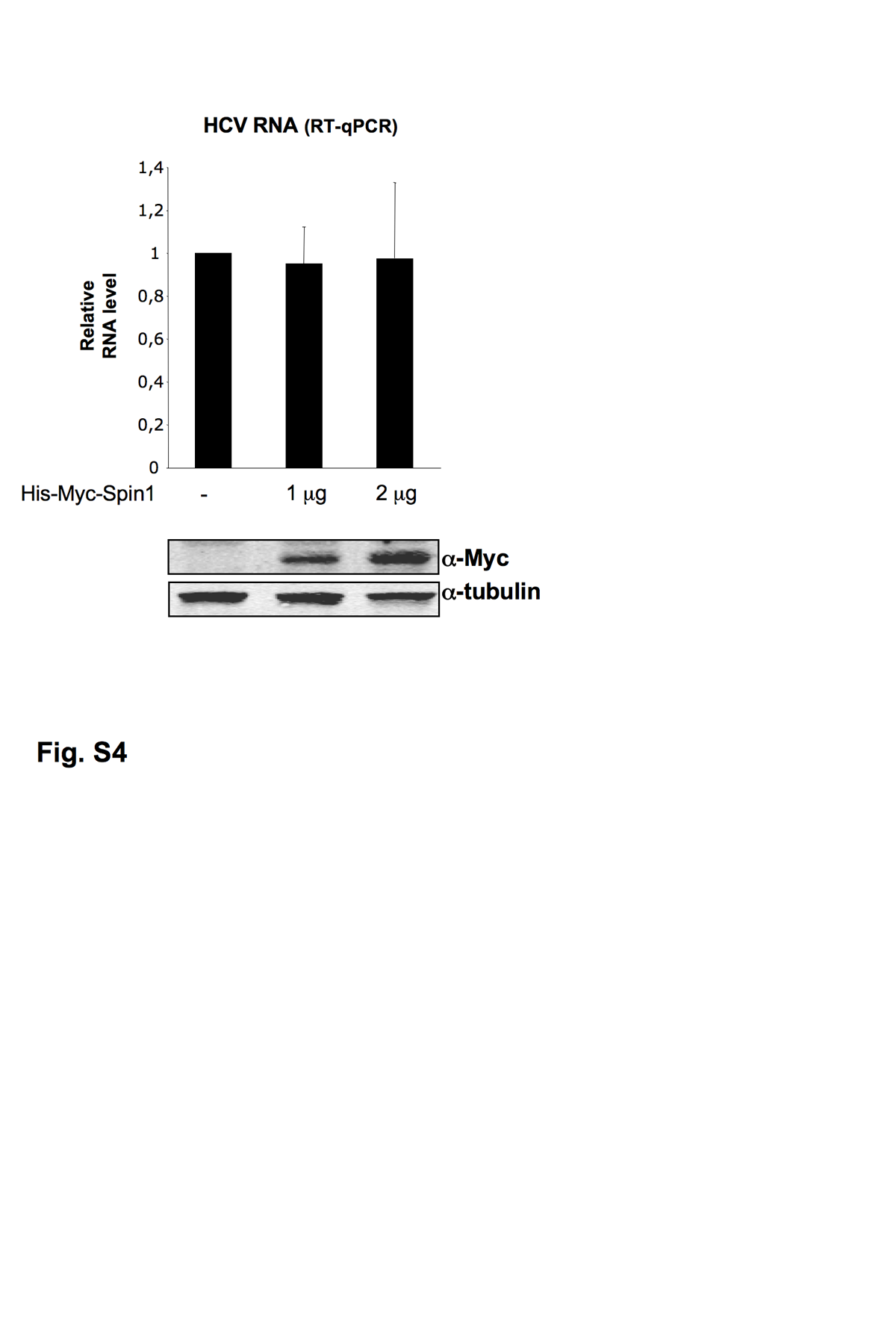

Supplement: Figure S4 — Huh7.25.CD81 cells transfected with 1 or 2 µg of plasmid coding for His-myc-Spindlin1 or with a control plasmid, were infected with HCV at MOI 0.3. 48 h after infection, cells were collected for RNA extraction. Viral RNA was quantified by RT-qPCR. Spindlin1 expression was analyzed by immunoblotting with anti-Myc antibodies. (TIF) [file ppat.1004343.s004.tif]
