# Supplementary figures and images for: Correction: The Tudor Domain Protein Spindlin1 Is Involved in Intrinsic Antiviral Defense against Incoming Hepatitis B Virus and Herpes Simplex Virus Type 1
Source: PLoS Pathog. 2020 Dec 11;16(12):e1009135. doi: 10.1371/journal.ppat.1009135 (PMC7732092; doi:10.1371/journal.ppat.1009135)

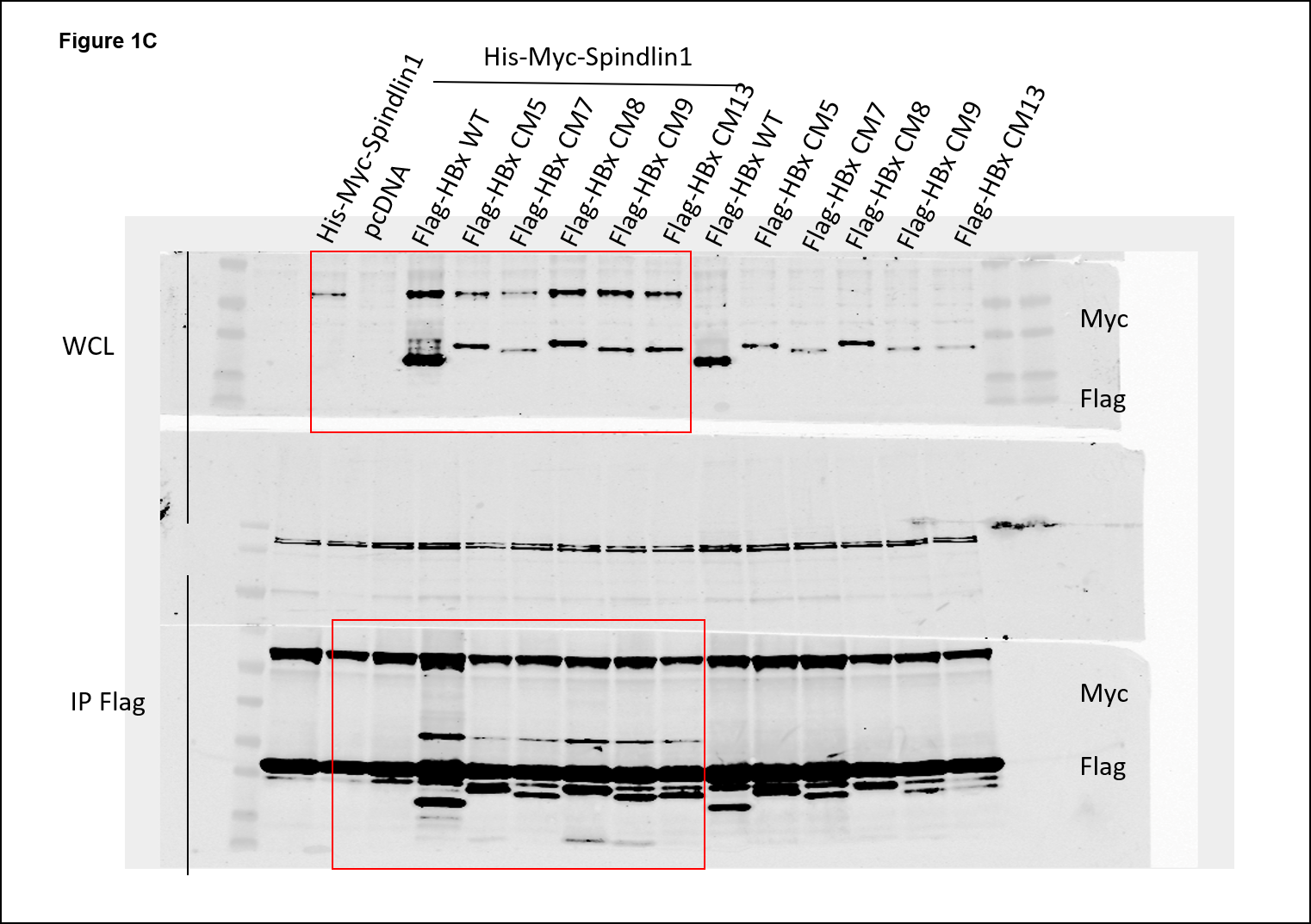

Supplement: S1 File — (TIF) [file ppat.1009135.s001.tif]

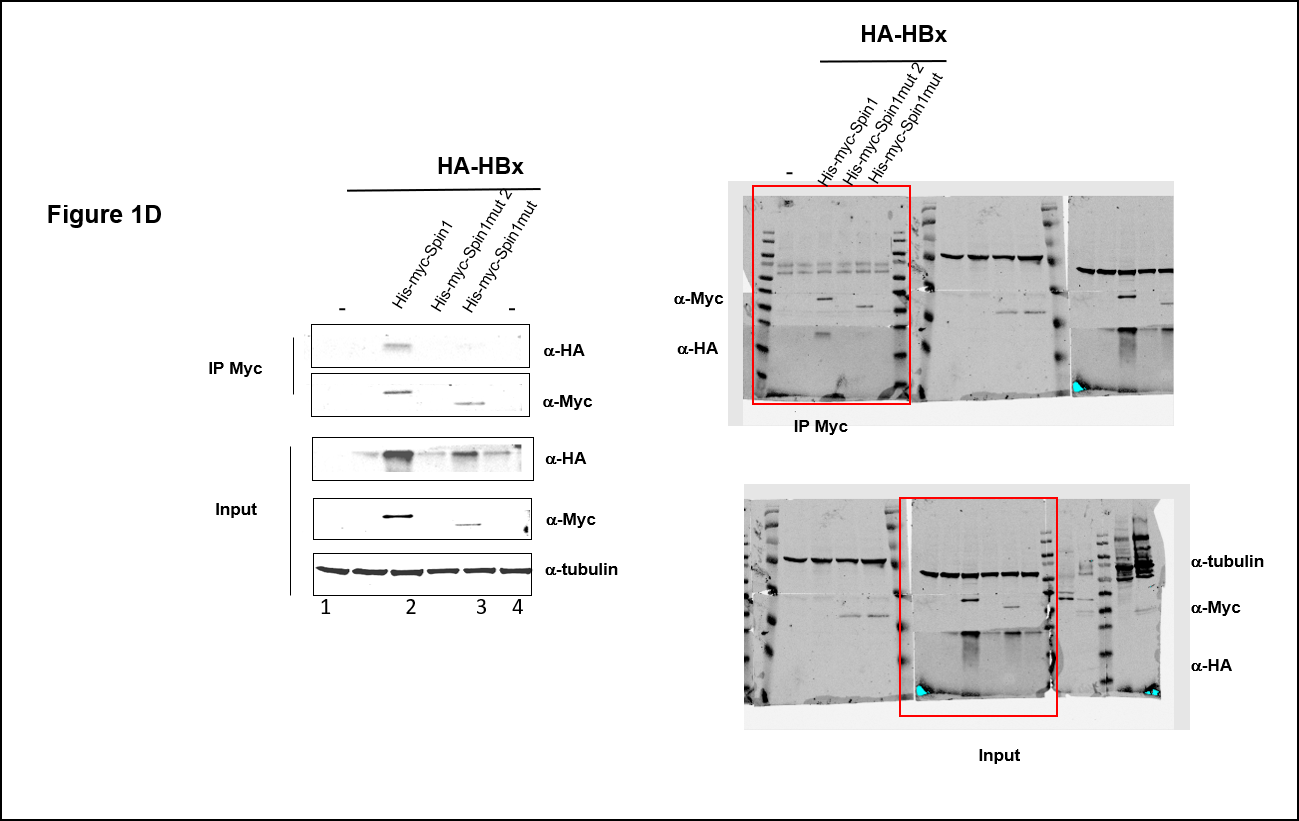

Supplement: S2 File — (TIF) [file ppat.1009135.s002.tif]

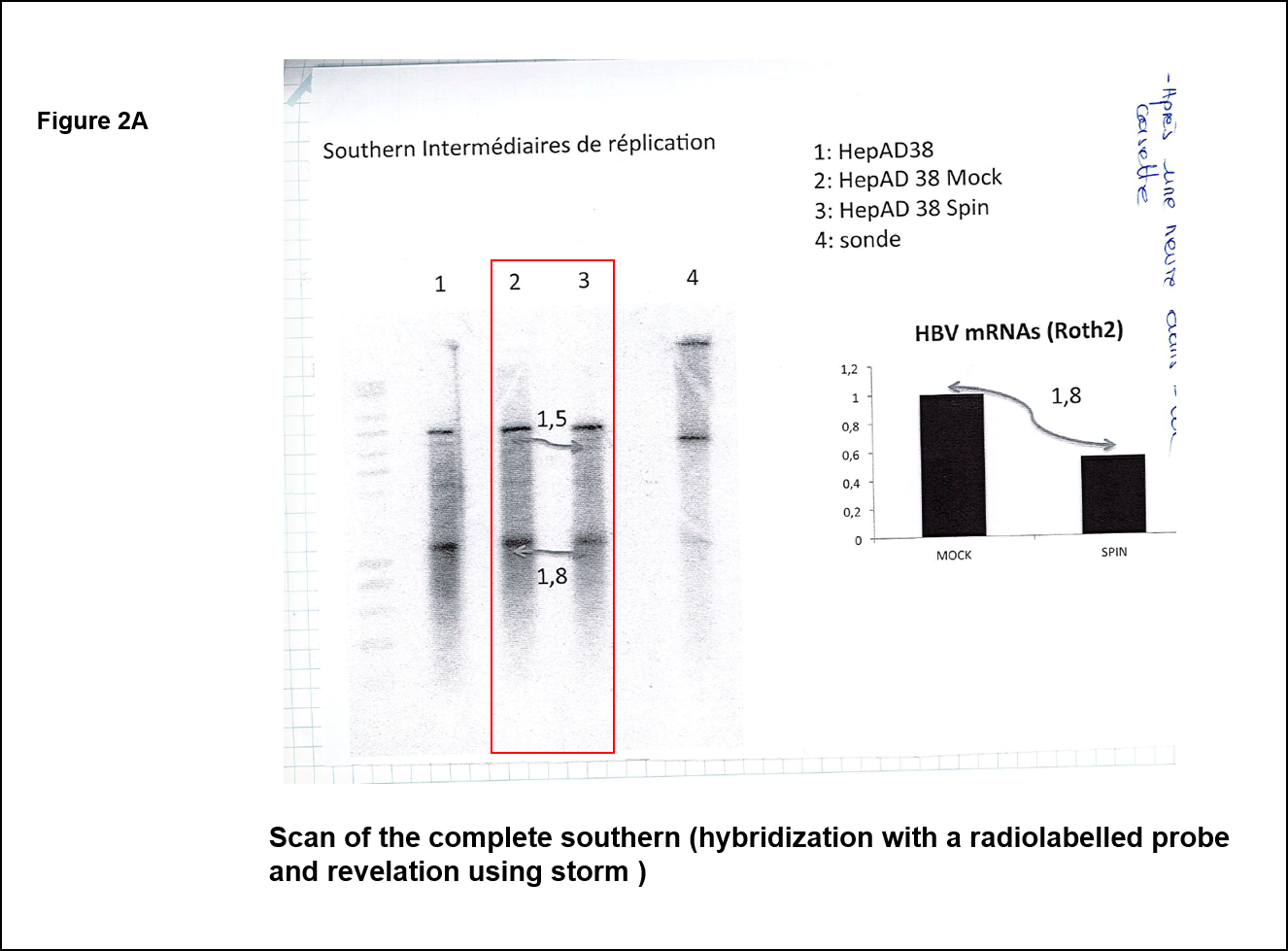

Supplement: S3 File — (TIF) [file ppat.1009135.s003.tif]

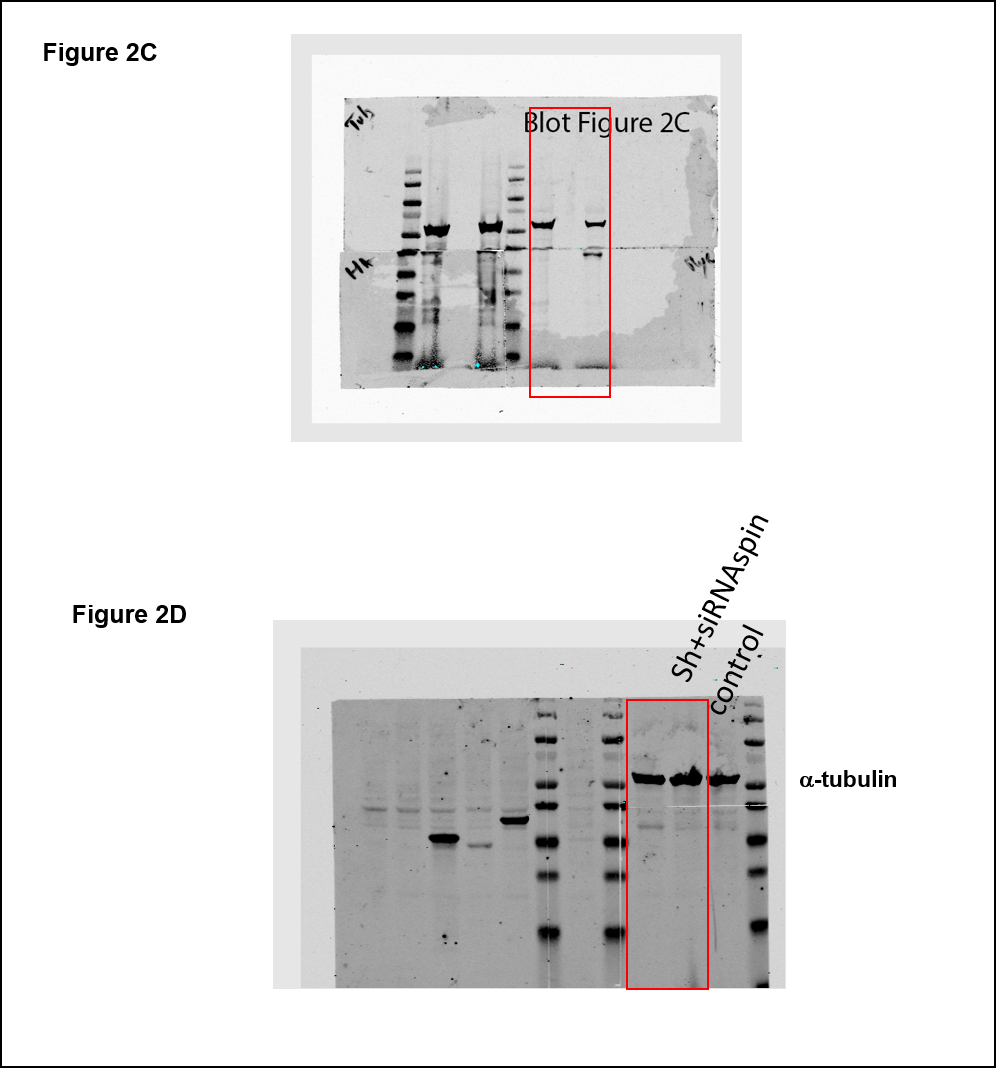

Supplement: S4 File — (TIF) [file ppat.1009135.s004.tif]

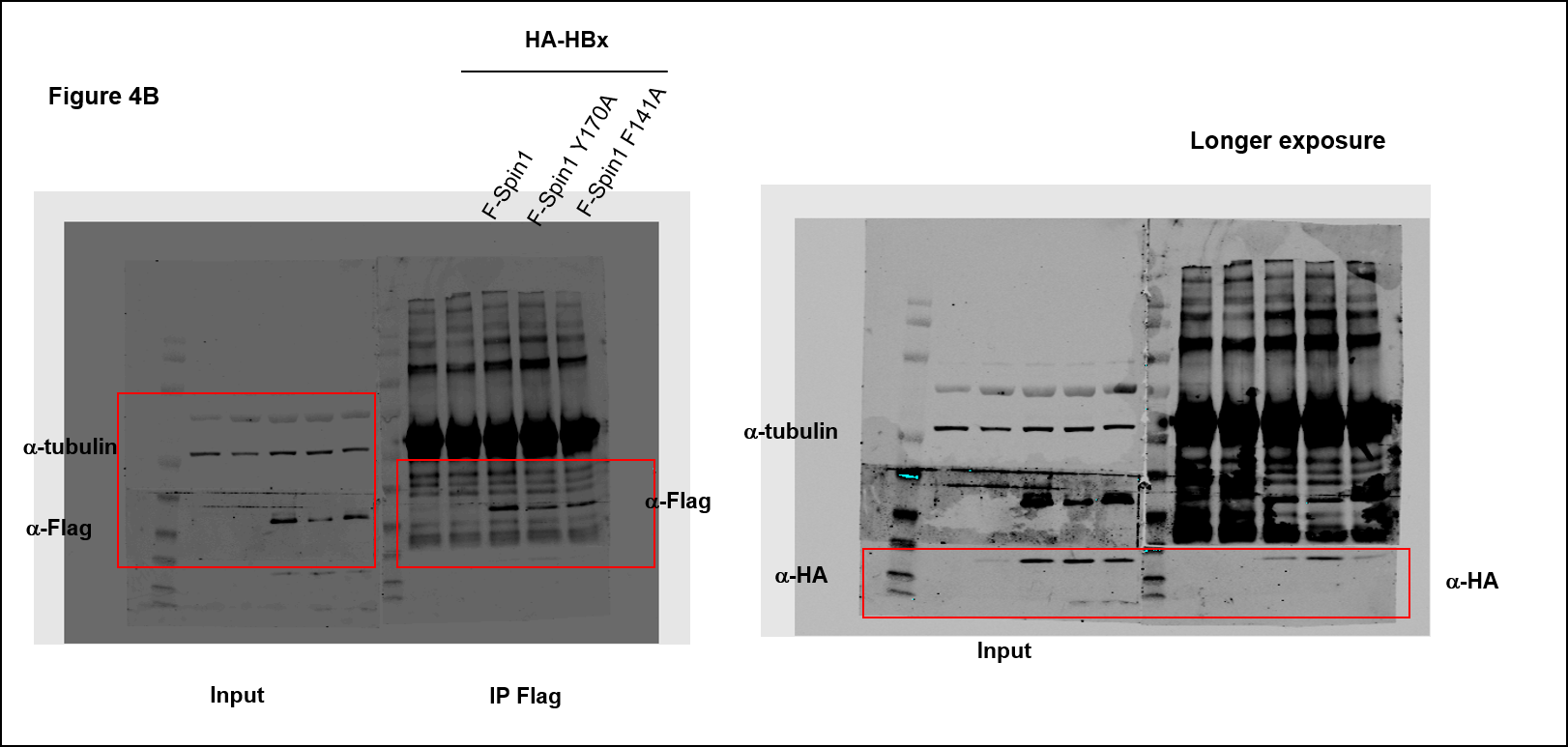

Supplement: S5 File — (TIF) [file ppat.1009135.s005.tif]
